# An immune memory–structured SIS epidemiological model for hyperdiverse pathogens

**DOI:** 10.1073/pnas.2218499120

**Published:** 2023-11-01

**Authors:** André M. de Roos, Qixin He, Mercedes Pascual

**Affiliations:** ^a^Institute for Biodiversity and Ecosystem Dynamics, University of Amsterdam, Amsterdam 1090 GE, The Netherlands; ^b^Santa Fe Institute, Santa Fe, NM 87501; ^c^Department of Biological Sciences, Purdue University, West Lafayette, IN 47907; ^d^Department of Ecology and Evolution, The University of Chicago, Chicago, IL 60637

**Keywords:** malaria parasite, tipping point, alternative steady states, structured population model, multigene family

## Abstract

A hyperdiverse class of pathogens of humans and wildlife, including the malaria parasite *Plasmodium falciparum*, relies on multigene families to encode antigenic variation. As a result, high (asymptomatic) prevalence is observed despite high immunity in local populations under high-transmission settings. The vast diversity of “strains” and genes encoding this variation challenges the application of established models for the population dynamics of such infectious diseases. Agent-based models have been formulated to address theory on strain coexistence and structure, but their complexity can limit application to gain insights into population dynamics. Motivated by *P. falciparum* malaria, we develop an alternative formulation in the form of a structured susceptible-infected-susceptible population model in continuous time, where individuals are classified not only by age, as is standard, but also by the diversity of parasites they have been exposed to and retain in their specific immune memory. We analyze the population dynamics and bifurcation structure of this system of partial-differential equations, showing the existence of alternative steady states and an associated tipping point with transmission intensity. We attribute the critical transition to the positive feedback between parasite genetic diversity and force of infection. Basins of attraction show that intervention must drastically reduce diversity to prevent a rebound to high infection levels. Results emphasize the importance of explicitly considering pathogen diversity and associated specific immune memory in the population dynamics of hyperdiverse epidemiological systems. This statement is discussed in a more general context for ecological competition systems with hyperdiverse trait spaces.

## Significance

As an immune evasion strategy under high transmission, important pathogens of wildlife and humans, including *Plasmodium falciparum* malaria, rely on vast diversity of genes encoding surface proteins targeted by the immune system. The large number of resulting “strains” (different gene combinations in pathogen genomes) challenges application of standard epidemiological models. Motivated by the malaria parasite, we present and analyze a modeling framework that tracks gene diversity explicitly together with immune memory of previous exposure. Dynamics exhibit alternative equilibria of high and low prevalence and an associated tipping point as transmission decreases. Thus, a sharp drop in prevalence can occur with intervention, but only under a drastic reduction in pathogen diversity to prevent rapid rebound of infection levels in the population.

Many pathogens from bacteria to fungi to protozoa, including the malaria parasite *Plasmodium falciparum*, rely on vast and evolving antigenic variation to evade host adaptive immunity under high transmission intensity (1). Strain hyperdiversity challenges the application of established mathematical models for the population dynamics of infectious diseases in the form of systems of ordinary differential equations, extensions of the well-known SIR (susceptible-infected-recovered) model to incorporate a fixed and typically low number of types.

Hyperdiverse pathogens of humans and wildlife encode variant surface antigens (VSA) with multigene families (2). As an example, *P. falciparum* in high-transmission regions of Africa carries 50 to 60 gene copies of the *var* gene family, which encodes for the major antigen of the blood stage of infection, the protein known as *Pf*EMP1 (for *P. falciparum* Erythrocyte Membrane Protein 1) (3). This protein is exported to the surface of infected erythrocytes where it helps cytoadherence to microvasculature but is exposed to the immune system (4). Successive and coordinated expression of *var* genes during blood stage proliferation reduces immune clearance by the host and enables long chronic infection (5). Thousands to tens of thousands of different *var* gene types have been documented in local parasite populations on the basis of nucleotide sequence divergence (6, 7). Vast combinatorial diversity ensues in the *var* gene repertoires carried by different parasite genomes. These repertoires exhibit extremely limited overlap between any two parasite genomes under high transmission, despite high rates of recombination in the sexual stage within the mosquito vector (7). High recombination rates have raised questions on the plausibility of strain structure from immune selection, the negative frequency-dependent selection arising from adaptive immunity (8–12). In addition, novel gene variants are constantly generated through mitotic recombination and mutation in this complex epidemiological system (13, 14). Examples of other multigene families encoding large antigenic variation can be found in other protozoa of vector-borne transmission (e.g., *vsg* in *Trypanosoma brucei*) and also in bacteria and fungi of direct transmission (e.g., *pil* in *Neisseria meningitidis* and *msg* in *Pneumocystis carinii*) (2).

Large antigenic diversity enables immune evasion and therefore high asymptomatic prevalence in host populations despite repeated exposure of individuals to the pathogen throughout their life (15). Although previous exposure confers protection against clinical disease (16), it does neither prevent reinfection nor preclude concurrent infection by different parasites known as superinfection (17). Asymptomatic falciparum malaria prevalence as high as 40 to 80% has been documented in high-transmission endemic regions of Sub-Saharan Africa across age classes, with individuals characteristically carrying multiple distinct parasites concurrently (e.g., ref. 18). The result is a large reservoir of infection for the mosquito vector to transmit the pathogen, which opposes intervention and elimination efforts. Such epidemiological characteristics lie at the opposite extreme from those of SIR dynamics, whereby a single exposure, or a low number of exposures, can provide complete protection to both clinical disease and reinfection, giving rise to the central phenomenon of herd immunity. The well-understood nonlinear and epidemic dynamics of SIR, SIRS systems, and their seasonal counterparts, do not extend to hyperdiverse pathogens and their epidemiological characteristics. Multiple variations in immunity representation exist in models that consider boosting by repeated exposure (e.g., ref. 19) and reinfection and superinfection (e.g., refs. 20 and 21), but these formulations consider only generalized protection that does not depend on parasite diversity explicitly.

Agent-based models (ABMs) that track each individual host and its specific memory of previous infections have been formulated to model transmission dynamics in a way that incorporates the combinatorial genetic diversity of the malaria parasite underlying antigenic variation (10, 11, 22, 23). These computational stochastic models have contributed to the development of theory on parasite population structure and negative frequency-dependent selection, including an incipient understanding of their role in high resilience to intervention (23, 24). In particular, the importance of the positive feedback between transmission intensity and strain diversity has been emphasized, with the suggestion of hysteresis in response to reduced vs. increased transmission (23). The complexity of ABMs limits however analytical investigation. Equation-based models more amenable to analysis have explicitly considered pathogens structured by genetic similarity (e.g., refs. 25 and 26). These models have the advantage of tracking the number of infected and recovered individuals for specific strains but have the disadvantage of primarily only being tractable when considering simplified assumptions on cross-immunity toward a small set of similar strains. Their application to large antigenic diversity remains problematic. Here, we present a complementary equation-based modeling framework that relaxes explicit consideration of specific strains but considers the local pool of antigen-encoding genes by incorporating host population structure as a function of both individual age and memory of past infections. This system of partial-differential equations further explicitly tracks total pathogen diversity. We show that the positive feedback between pathogen diversity and the force of infection leads to the existence of alternative steady states and an associated tipping point with changing transmission rates. This bifurcation structure sustains high prevalence and the return to high prevalence upon perturbation, unless pathogen diversity is drastically decreased.

To model the epidemiological dynamics of malaria we combine an age-structured model of susceptible and infected hosts (27) with previously developed models of superinfection (21, 28) (*Materials and Methods*). As a unique feature, our model also tracks the number (but not the identity) of different parasite genes the hosts of a particular age have encountered previously. Besides age-structured, the model is hence also immunity-structured, as hosts build up a memory of previous infections during their life (see *Materials and Methods* for details). Hosts may become infected through transmission events, the frequency of which is proportional to a transmission intensity parameter, the contact rate $k_{0}$. Infection probability furthermore depends on the frequency of infected individuals in the host population. During transmission events, hosts are assumed to be exposed to a fixed number of parasite genes (a repertoire) that are randomly selected from the total parasite gene pool. Assuming that hosts are immune against the VSA encoded by previously encountered parasite genes, a transmission event leads to successful infection if at least one of the parasite genes in the repertoire has not been encountered previously, while the duration of infection increases (the recovery rate decreases) with the number of genes in the repertoire that have not previously been encountered (*Materials and Methods*). The number of unique parasite genes in a repertoire is determined by the diversity in the total parasite gene pool, which itself is dynamic. Parasite diversity increases through mitotic recombination of existing genes in an infection as well as through introduction of new genes by immigrating hosts carrying an infection (*Materials and Methods*). Key features of the model are an inclusion of an explicit equation for the rate of change in genetic diversity, and the tracking of the fraction of this changing diversity a host has been exposed to. For simplicity, we consider here that different genes encode different VSAs. We also refer to gene diversity with the understanding that this maps to antigenic diversity, an aspect we later discuss. We also subsumed transmission through the vector in a contact rate parameter (as, for example, in ref. 27) without loss of generality, as we also discuss. We refer to this parameter hereafter as transmission potential. For vector-borne transmission under certain assumptions, it can be interpreted as the vectorial capacity, defined as the rate at which future potential inoculations arise from a current infectious host.

**Fig. 1.**
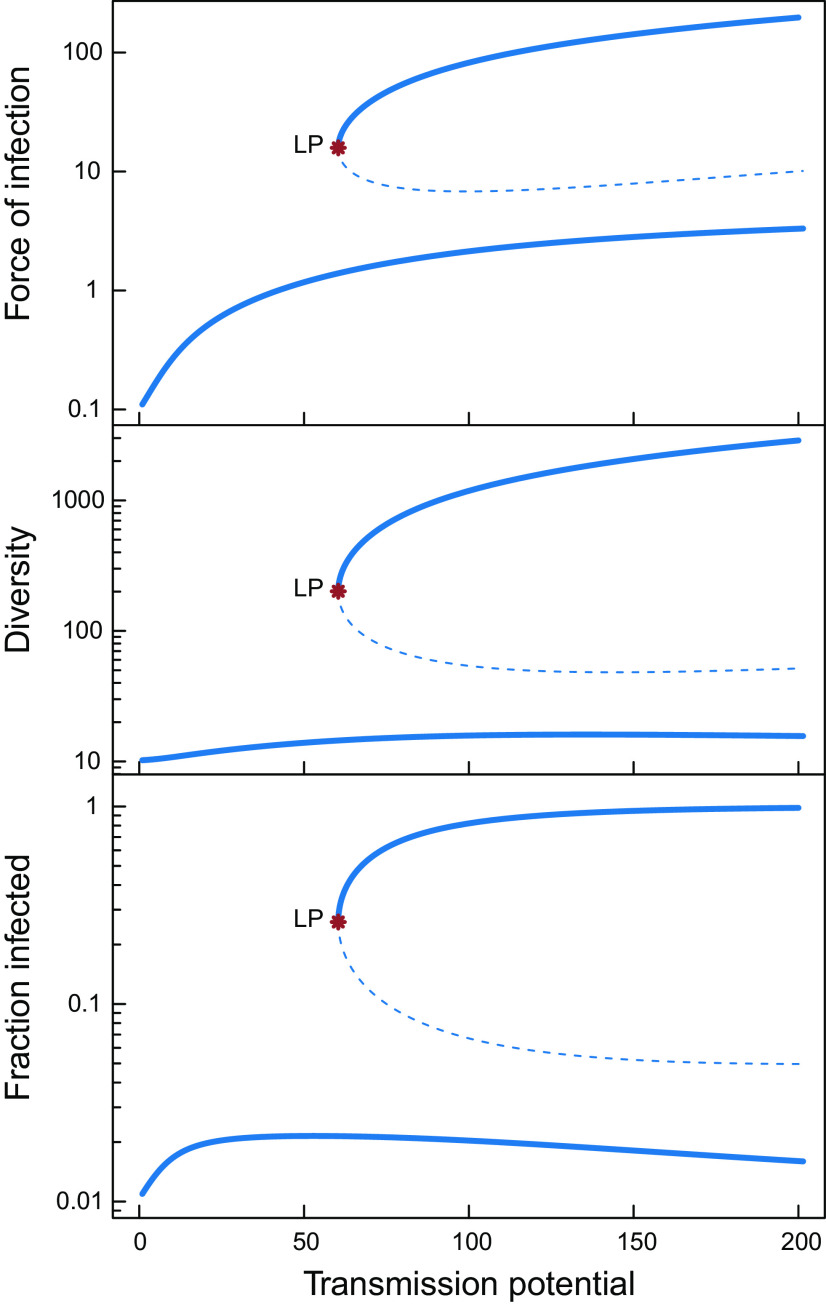
Equilibrium states of the malaria model as a function of the transmission potential, the contact rate parameter $k_{0}$ (per year). Solid and dashed lines refer to stable and unstable equilibrium states, respectively. The location of the tipping point (saddle–node bifurcation point) is marked as LP.

For varying levels of transmission potential, as parameterized by this contact rate $k_{0}$, our immunity-structured model predicts co-occurrence of two types of alternative equilibrium states (Fig. 1, *Materials and Methods*, and *SI Appendix* for details on computing equilibrium states). For default parameters, a low-prevalence equilibrium state occurs for all values of the transmission intensity, in which the force of infection, the parasite gene pool diversity, and the fraction of infected host individuals are all low. Parasite diversity in this state is mostly determined by immigration of infected individuals. Above a threshold level of transmission potential, however, a high-prevalence equilibrium state occurs, in which the majority of host individuals are infected and both the force of infection as well as the parasite gene pool diversity are one to two orders of magnitude larger than those in the low-prevalence equilibrium. Numerical simulations of the model reveal that both equilibrium states are stable (*Materials and Methods* and *SI Appendix*). An unstable equilibrium (saddle point; dashed lines in Fig. 1) separates the two stable equilibrium states from each other.

**Fig. 2.**
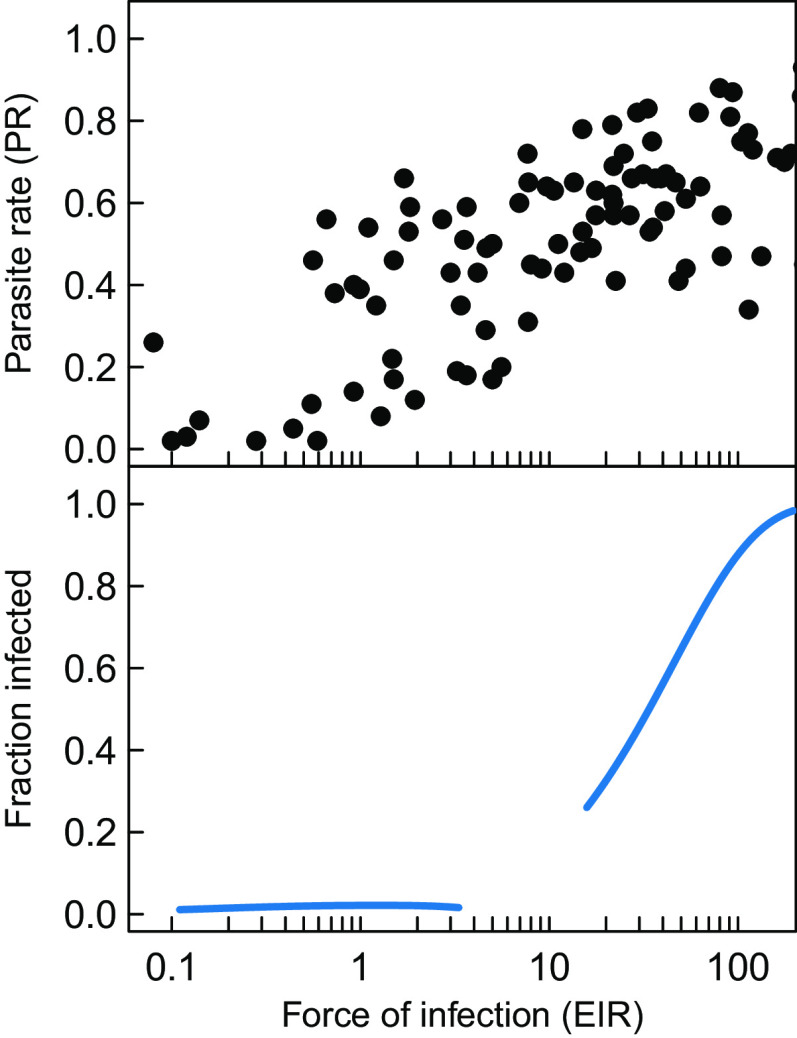
*Top*: Observations of the parasite rate and the entomological inoculation rate (EIR) from ref. 29. *Bottom*: The relationship between the fraction of infected individuals in the population and the force of infection in the stable equilibrium states observed for different values of the transmission potential (Fig. 1). In the model, EIR was computed as the force of infection λ for a naive susceptible individual.

In the low-prevalence equilibrium, the fraction of infected hosts is virtually independent of the force of infection, whereas the fraction of infected hosts increases with the force of infection in the high-prevalence equilibrium (Fig. 2). Epidemiological data often show a sigmoidal relationship between the fraction infected, also referred to as the parasite rate (PR), and the force of infection, measured as the entomological inoculation rate (EIR) (29, 30). The data presented in ref. 29, for example, show that for EIR values below 1, the parasite rate tends to be rather constant and below 10%, whereas for EIR values larger than 10, the parasite rate tends to be larger than 40% and increasing with EIR (Fig. 2). The model results show roughly similar relationships between PR and EIR in these two regimes, although our model is completely deterministic and hence does not show any variation around the PR-EIR relationship. In between these two regimes, the epidemiological data tend to be highly variable with PR ranging from 10 to 70%, whereas the model predicts no stable equilibrium states to occur with these combinations of PR and EIR (Fig. 2). We suggest that the observed variability in the data in this range of EIR values may in fact be indicative of the absence of a stable equilibrium state and that the highly variable observations in this range arise from stochastic or time-dependent variability in transmission.

**Fig. 3.**
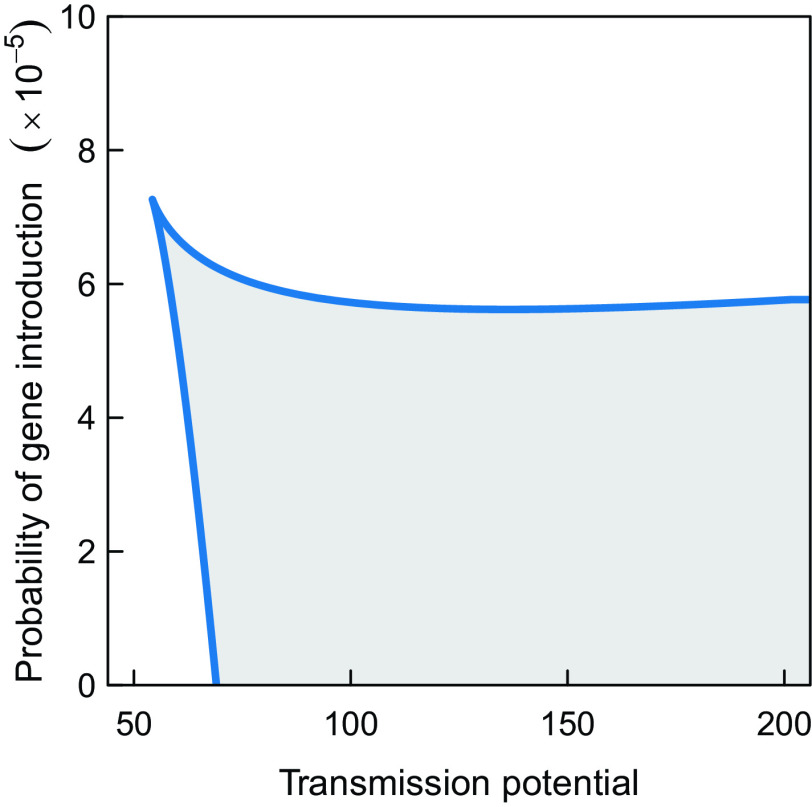
Parameter domain (*gray*) for which alternative stable equilibrium states occur as a function of the probability that an immigration event leads to the introduction of a new parasite gene (parameterized in the model by $P_{I}$) and the transmission potential, the contact rate parameter $k_{0}$.

The two alternative equilibrium states co-occur when the transmission potential is above a threshold level and the probability that immigration of infected hosts leads to an increase in the parasite diversity is low (Fig. 3). When the probability that immigration of infected hosts leads to an increase in the parasite diversity is higher, alternative stable equilibrium states no longer occur, but the model still predicts a sharp transition between a parameter regime with a low- and a high-prevalence equilibrium state (*SI Appendix*, Fig. S1).

**Fig. 4.**
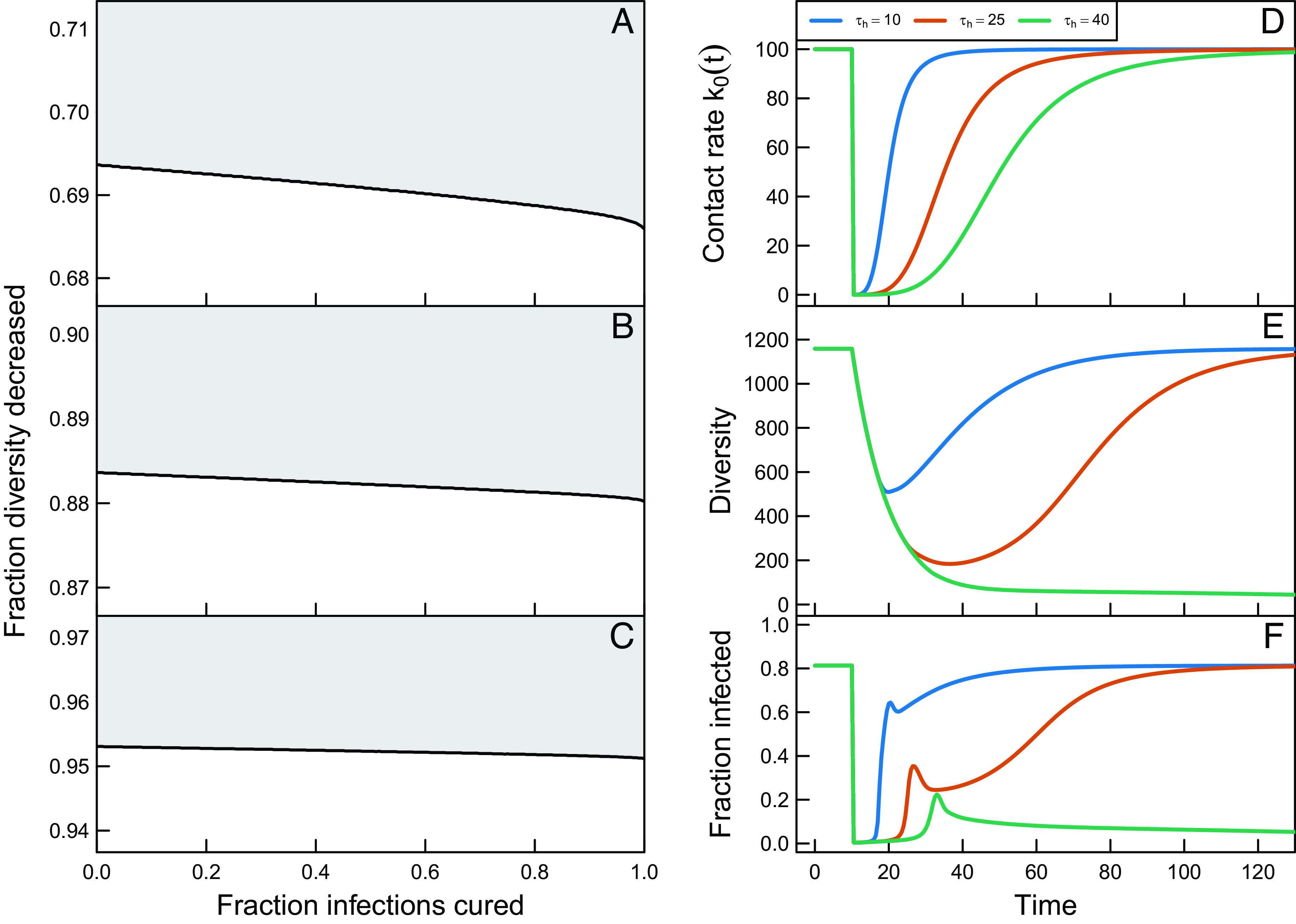
Combinations of the fraction of infected individuals to be cured and the reduction in diversity that is required to change the population from the high-prevalence state to the low-prevalence state for contact rates $k_{0}=65,75$ and 100 (per year) (*A*–*C*, respectively). *Right*: Changes in the contact rate $k_{0}\left(t\right)$ (*D*), parasite gene pool diversity (*E*), and the fraction of the individuals that are infected (*F*) following a temporary and transient intervention, described by the time-dependent function $k_{0}\left(t\right)=100{\left(\left(t-10\right)/\tau_{h}\right)}^{4}/\left(1+{\left(\left(t-10\right)/\tau_{h}\right)}^{4}\right)$.

The high-prevalence equilibrium state is very resilient against perturbations and drastic interventions are required to induce a shift of the host population from the high-prevalence equilibrium state to the low-prevalence equilibrium state. We assessed resilience of the high-prevalence equilibrium state by analyzing whether or not the host population would return to the high-prevalence state when a random fraction of infected host individuals was cured of the disease and parasite diversity was independently reduced by a particular fraction. With these computations, we obviously ignore the fact that from a practical viewpoint such independent manipulation of the fraction of infected hosts and the parasite diversity is largely unrealistic, as the latter would generally accompany parasite clearance from hosts via drug treatment and other interventions reducing prevalence. Nevertheless, a shift from a high-prevalence to a low-prevalence equilibrium state can only be achieved by a significant reduction in parasite diversity, whereas there is only a minor effect of the fraction of infected host individuals that is cured (Fig. 4). If $k_{0}$ is slightly increased above the threshold for the high-prevalence equilibrium, a reduction of at least 65% in parasite diversity is already required for the system to switch to the low-prevalence equilibrium (Fig. 4*A*), whereas for higher values of $k_{0}$, parasite diversity has to be decreased by 90% or more to induce such a shift (Fig. 4 *B* and *C*).

Similarly, a temporary reduction in contact rates only leads to a shift from the high-prevalence to the low-prevalence equilibrium state if the reduction in transmission intensity lasts sufficiently long, roughly a period equal to the average lifespan of the host individuals (Fig. 4*D*–*F*). The transient dynamics during and following such a temporary reduction in transmission emphasize the important role of parasite diversity in disease dynamics: The fraction of infected host individuals drops almost immediately to very low levels when $k_{0}$ is reduced (Fig. 4*F*), whereas parasite diversity decreases much more slowly (Fig. 4*E*). When transmission reduction measures are relaxed, the fraction of infected host individuals rapidly increases to an intermediate level, which is followed by a much slower increase to the original infection levels. This second phase of recovery is on a similar time scale as the recovery of parasite diversity and reveals the feedback between parasite diversity, host immunity, and the force of infection, which plays a central role in the dynamics of the system.

In both equilibrium states, the highest infection levels occur among very young hosts, yet the specific age-prevalence curves differ (*SI Appendix*, Fig. S2). Specifically, in the low-prevalence equilibrium infection levels never exceed 30% at any age, whereas in the high-prevalence equilibrium, virtually all young hosts are infected, while the infection levels never drop below 80% among older hosts. Furthermore, superinfection almost never occurs in the low-prevalence equilibrium, whereas in the high-prevalence equilibrium superinfection is common, in particular, among young hosts. Remarkably, though, the fraction of the total parasite gene pool that hosts have encountered from birth up to a specific age does not differ between the two types of equilibrium states, despite the large difference in parasite diversity in the two states. This fraction rapidly increases with age to an asymptotic level of around 0.9 (*SI Appendix*, Fig. S2). The similar age dependence of this fraction in the two states suggests that the build-up of immunity during life is not crucial for the occurrence of the two alternative equilibrium states. Based on this observation, we derived a simplified version of the immune memory–structured SIS model, in which we assume that the fraction of the parasite gene pool previously encountered by host individuals is equal to a constant p=0.9 irrespective of their age. Furthermore, we drop the assumption of a finite lifespan of the host. The resulting model is specified in terms of two ordinary differential equations for the fraction of infected individuals in the entire population and the total diversity of the parasite gene pool (*SI Appendix*; from here referred to as the simplified ID model). Analogous to our original model, this simplified ID model also predicts the occurrence of alternative stable equilibrium states over similar ranges of parameter space as the full model (compare *SI Appendix*, Fig. S3 with Figs. 1 and 3). This correspondence between our original model and its simplified analog suggests that the increase in parasite diversity due to the generation of new parasite genes in infected hosts is the key mechanism underlying the alternative stable states and the separation between distinct domains with low and high parasite prevalence.

Despite the similarity regarding alternative stable equilibrium states, qualitative and quantitative properties of the transient dynamics differ markedly between the ID model and the age- and immune memory–structured one, as perturbations act in the latter via population distributions. The simplified ID model predicts that the high-prevalence equilibrium state is much more resilient to perturbations and that reductions in diversity larger than 85% rather than 65% (cf. *SI Appendix*, Fig. S4 and Fig. 4) are required to induce a shift to the low-prevalence equilibrium state, even for values of the contact rate $k_{0}$ close to the tipping point. Furthermore, the simplified ID model predicts that the dynamics following a temporary reduction in the transmission potential lead to an almost instantaneous return to the high-prevalence equilibrium state or an instantaneous shift to the low-prevalence equilibrium state for short and long duration of the reduction in transmission potential, respectively (cf. *SI Appendix*, Fig. S4 and Fig. 4). The rapid return to a high level of infected host individuals seems largely independent of the slower recovery of the parasite diversity. In contrast, the age- and immune memory–structured model shows both parasite diversity and the fraction of infected host individuals to increase at a similar and longer timescale following a temporary reduction in the bifurcation parameter (cf. *SI Appendix*, Fig. S4 and Fig. 4) as a result of the interplay with the dynamics of host immunity building up over a host’s lifetime. The fraction infected also exhibits an overshoot that is missing in the simpler model, reflecting changes in the distribution of immune memory in the population following intervention.

Together, both the simplified and immune memory–structured models allow us to demonstrate bistability arising from a fundamental positive feedback between transmission intensity and the size of the local pool of diversity underlying strain variation. We have shown that this emergent process can introduce a critical transition to regimes with alternative steady states. Implications of such dynamics include sharp variation in equilibrium prevalence with changes in transmission intensity and more complex transient responses, where small differences in intervention can lead to a rebound or not of the prevalence. Importantly, our results demonstrate that the key variable for the resilience of the system is parasite diversity and that only a drastic reduction of this variable leads to the lower prevalence equilibrium. We note here that the bifurcation parameter that scales the contact rate via vectors, which we varied to examine equilibria, is “extrinsic” to the system and reflects suitability for transmission due to environmental and public health factors. These are factors affecting, for example, the habitat suitability of a region to mosquito vectors. By contrast, the force of infection or entomological inoculation rate is emergent from the dynamics as it includes the prevalence of infection in the human (and mosquito) population.

In practice, parasite diversity in malaria and other hyperdiverse pathogens can only be reduced indirectly by interventions that decrease transmission. Together with the demonstrated extreme reduction in diversity required to reach the low-prevalence equilibrium, this fact severely limits, if not prohibits, the likelihood of observing in nature the low-prevalence alternative state in areas where contact rates are above the critical point. Thus, a main implication of bistability in this system is not the actual observation of alternative steady states, but the introduction of a critical point, and therefore a threshold, in contact rates, at which prevalence changes drastically. (This transition is also the behavior that remains when immigration is sufficiently high so that bistability itself is no longer present). Two major empirical observations in malaria epidemiology are nevertheless consistent with the existence of the transition we have described. The first one concerns the resilience of high-transmission regions to control efforts, whereby the relaxation of transient strong interventions has been typically followed by the rapid rebound of the system to originally high prevalence levels (31–33). The second one concerns what has been called the “stability” or “stickiness” of the malaria-free state in locations where the disease has been eliminated, despite parasite reintroductions and immigration-driven low prevalence (34, 35). By contrast to the rapid resurgence of prevalence in most locations that have failed to eradicate, the elimination state has persisted in a large fraction of countries that have achieved elimination. A number of possible explanations have been discussed including the establishment of well-developed health systems; bistability constitutes an additional one. Most such countries started however with low-to-moderate transmission. An interesting example of high transmission potential is provided by the island state of Mauritius, with a long history of elimination efforts and a successfully sustained malaria-free state since 1998 (36). The country maintains an active program for the prevention of re-establishment (POR) of malaria including surveillance of travelers from endemic regions, vector management, and a strong healthcare system. It is described nevertheless as a place with high malaria “receptivity” (36), a concept introduced during the Global Malaria Eradication Programme (GMEP) era, with varied definitions but a commonly accepted meaning of the degree to which a location supports local malaria transmission (37). Our results underscore the central importance of keeping the importation of diverse strains from happening.

The existence of the threshold we propose here opens the door for investigating indicator quantities related to parasite diversity that would signal the approach to a drastic transition in prevalence. For increased realism, this avenue could be pursued with extensions of the model in the form of agent-based formulations (ABMs). We expect our results to provide a basis for comparison with the dynamics of detailed stochastic ABMs concerning responses to intervention efforts. Prior research has shown the advantage of synthesizing results from the two approaches: The abstract PDE formulation provides a more complete inventory of the dynamic patterns that can occur in ABMs and also informs potential cycles initiating from unstable steady states in the ABM (38). The difference in temporal trajectories with decreasing vs. increasing transmission described in Holding et al. (23) as possible hysteresis or delayed responses, is of interest in this regard as suggestive but not conclusive of bistability. Further investigation is required, including longer time horizons.

Previous mathematical models of malaria transmission considering generalized but not specific immunity have shown that superinfection and reinfection can both lead to alternative steady states (20, 21, 27). Nevertheless, the mechanism here behind the critical transition differs and concerns explicit antigenic diversity of the pathogen. This lends further support to molecular surveillance for intervention efforts in high-transmission regions based on the part of the genome that encodes such diversity (e.g., ref. 33)

The lower equilibrium in our models differs from a disease-free one because we have considered an open system, with a contribution to the force of infection from outside the local population from immigration. Although this setup is realistic, it impedes consideration and analysis of a disease-free equilibrium at low levels of transmission intensity, in the traditional sense of invasibility of infection at a threshold value of $R_{0}$. For this purpose, we can modify the PDE model to remove immigration of infection. In order to maintain D above a given minimum value, we specify the loss rate of diversity in a diversity-dependent manner. This value is interpreted as the minimum number of distinct antigen-encoding genes in an invading parasite at low transmission (varied from 1 to L; see *SI Appendix*, Fig. S5 for details). Such a minimum is biologically realistic and was necessary to approach the disease-free equilibrium in a meaningful manner, as our original formulation assumed that D remained at least above one. We confirmed that bistability remains for sufficiently high contact rates. For lower rates, we now have an additional bifurcation, a transcritical one, at which the positive lower equilibrium loses stability and exchanges it with the disease-free one (*SI Appendix*, Fig. S5). The existence of two bifurcation points is robust to changing the imposed lowest value of diversity, with this value determining the transmission potential at which disease invades (*SI Appendix*, Fig. S6).

As for any complex system involving diversity, the SIS model we presented here is necessarily a simplification of biological reality. It was developed as an equation-based simplification of agent-based models addressing strain structure in malaria (e.g., refs. 10 and 11). It was inspired by the hyperdiversity of *P. falciparum* in high-transmission endemic regions, from the perspective of the multigene family that encodes the major antigen of the blood stage of malaria infection. Additional epidemiological considerations that can be incorporated into this framework for future application to malaria include 1) effects of generalized immunity representing protection from infection conferred from more conserved antigens (e.g., the current vaccine targets), 2) seasonality of transmission, and 3) explicit vectors. The implicit treatment of transmission via vectors with a parameter akin to vectorial capacity (39) assumes fast population dynamics of infection in the mosquito relative to those in the host and that total mosquito abundance is either constant or equilibrates rapidly relative to changes in the environment. These are standard assumptions in models from which vectorial capacity has been derived and defined in the first place (see ref. 40). We do not expect the explicit consideration of vectors to modify our results, as such an extension should only introduce distributed delays in transmission. More generally, the modeling framework should apply to other pathogens with large antigenic diversity encoded by multigene families, including those with direct rather than vector-borne transmission.

Key to our model is an explicit equation for pathogen genetic/antigenic diversity and the tracking of the fraction of this changing diversity a host has been exposed to. We have considered a detailed expression for the generation of novelty but not for its loss. For simplicity, we assumed this loss rate to be constant Eq. **11**, which is possible because the contribution to the force of infection from immigration implied that diversity never drops below a minimum level even at very low transmission intensity (Fig. 1). Our model hence considers an open system, which in our opinion is the most realistic setup for local malaria dynamics. We also explored invasion of a disease-free equilibrium in case of a closed population without any immigration, which requires however a change of the loss rate of diversity to a diversity-dependent formulation (see *SI Appendix* for details). This modified model exhibits the classical pattern of a stable, disease-free equilibrium at low transmission potential (low values of the contact rate $k_{0}$), exchanging stability with an endemic equilibrium with low diversity and prevalence when contact rate increases, which is the standard scenario of disease invasion typically associated with studies of $R_{0}$ (*SI Appendix*, Figs S5 and S6). Importantly, though, the bistability and the associated saddle–node bifurcation that occurs when contact rate is sufficiently high are unaffected by the change in model structure.

Moreover, the genetic composition of different strains is highly structured in that gene overlap between genomes is lower than the random expectation we have adopted here, as demonstrated with molecular data and deep sampling of *P. falciparum* populations in high transmission regions (7, 41). In particular, we expect that these considerations should further enhance the impact of diversity on transmission dynamics but not change the basic outcome.

As already stated, we considered the genetic diversity behind variant surface antigens (VSA) without making an explicit distinction between genes and their products. This is akin to the current study of strain structure from *var* genes, which defines different “types” on the basis of a given level in sequence divergence, and strains as repertoires of these types (6, 7). A better future understanding of genotype-phenotype maps in multigene families encoding antigenic variation would be extremely interesting and pertinent. Although the equilibrium values of D reached in our system are large, they are below those documented from deep sampling in field studies (6). We note however that D stands in the model for the common genes that coexist under immune selection. The large fraction of genes that are rare in empirical distributions from endemic regions (7, 11, 41) would therefore not be counted as part of this variable.

Interestingly, a parameterization of our model on the basis of the malaria literature, without any calibration, produces an intriguing similarity to the empirical relationship between prevalence rate and force of infection (Fig. 2). It is well known that stochasticity can interact with the bifurcation structure of deterministic models in ways that may explain the broad range of values observed in the transition from low to high prevalence. We note however that the EIR was computed in the model as the force of infection λ for a naive susceptible individual, which through its dependence on the contact rate parameter $k_{0}$ implicitly incorporates the transmissibility from an infectious bite to a human host (the probability that such an infectious bite actually results in an infection). Experimental estimates for this probability in mice range widely from about 0.1 to 0.8 (42) depending on the parasite load in the mosquito vector. The empirical EIR on the other hand does not include this probability but is likely biased toward lower values as the ELISA detection sensitivity in field measurements is limited by a minimum threshold of sporozoites (43). Since these two effects could cancel each other, we have presented here the direct comparison of EIRs without any corrections. Similarly, empirical estimates of disease prevalence may also be an underestimation due to submicroscopic infections. We did not adjust these values because submicroscopic infections are 1/3 as infectious as microscopic ones (44) and contribute a small fraction to the transmission except for low transmission regions around 20% where EIR is below 4 (45). Because we assumed uniform transmissibility among hosts, it is more appropriate to compare model prevalence to empirical microscopic prevalence. Despite these simplifications, the similarity between our model and empirical data is encouraging; it indicates that extensions of our model would be worth fitting to field data that include molecular epidemiology. Consideration of the part of the parasite genome under immune selection in transmission models applied to epidemiology should contribute to our understanding of dynamic transitions toward elimination.

More broadly, we can ask whether positive feedback between genetic diversity and intensity of ecological interactions (here competition of parasites for hosts) can lead to critical transitions in other hyperdiverse systems. The relevant diversity would encode here phenotypic traits that influence ecological interactions; these interactions would in turn influence individual fitness in a frequency-dependent manner. Theory in community ecology has begun to examine analytically models with such interactions through “environmental feedback” (46), and the well-known Janzen–Connell hypothesis makes such feedback central to species coexistence in rainforests (e.g., refs. 47 and 48). Although it is early to draw generalizations and analogies across different systems and levels of organization, the question of critical points in the capacity of systems to accumulate the genetic diversity necessary for hyperdiversity and resilience is intriguing. This question may be fundamental to the related but opposite goals of elimination in epidemiology and conservation in ecology.

## Materials and Methods

### Model Formulation.

We use an age-structured SIS model in terms of susceptible and infected individuals (27), assuming constant population size (see *SI Appendix* for detailed model derivation), such that it is only necessary to track the fraction s(t,a) of susceptible hosts at time t with age a. Unique features of our model are *i*) the introduction of a second individual-state variable besides age, to describe the specific immune memory individuals build during their lifetime from infection history; and *ii*) an explicit equation describing temporal changes in the overall genetic diversity of the parasite population encoding the variant surface antigens of interest.

We assume that hosts receive mosquito bites at a constant rate, independent of their age and infection status, with the force of infection λ(t) dependent on the frequency of infected individuals in the host population. We furthermore assume that migration of infected hosts increases the local force of infection by an amount $\lambda_{I}/N$ with N the total host population size, such that the force of infection is given by:

[1] $$\lambda \left(t\right)\;=\;k_{0}\frac{\mu }{1-\exp(-\mu A_{m})}\int_{0}^{A_{m}}\left(1-s(t,a)\right)\;e^{-\mu a}\;da+\frac{\lambda_{I}}{N}.$$

In this expression, μ represents the host mortality rate and $A_{m}$ its maximum age, while $k_{0}$ is a scaling constant, the contact rate.

During a biting event, a package of L genes encoding variant surface antigens is transferred, representing a given pathogen. For simplicity, we consider diversity in terms of genes and not their products (*Discussion*). These L genes are assumed to be randomly sampled from a pool of available diversity in the pathogen population, the total size of which we indicate with D. The expected number of unique genes delivered in a biting event is then given by:

[2] $$G=D\left(1-{\left(\frac{D-1}{D}\right)}^{L}\right)$$

(*SI Appendix*). Infection is assumed to occur when at least one of these unique genes has not been previously encountered by the host. To track immune memory, we introduce P(t,a) and p(t,a) (=P(t,a)/D) to represent the number of parasite genes and the fraction of the total parasite gene pool, respectively, that a host of age a at time t has previously encountered. The rate at which successful infections occur is then given by:

[3] $$\lambda_{s}\left(t\right)=\left(1-p{\left(t,a\right)}^{G}\right)\lambda \left(t\right),$$

while the rate at which new parasite genes are delivered during successful infection events is given by:

[4] $$G\left(1-p(t,a)\right)\lambda \left(t\right).$$

(see *SI Appendix* for the derivation).

Another parameter influenced by immune memory of the host is the recovery rate from the infected into the susceptible class. Its inverse is the duration of infection, whose value is key to parasite fitness. We adopt a well-known superinfection model by ref. 28 whereby temporally overlapping infections proceed independently from each other but influence the total duration of infection. We denote the duration of infection corresponding to the expression of one gene as $c_{0}$ and assume that the total duration decreases with the number of genes that the host has already encountered before since those genes are no longer expressed. The total duration of infection is therefore proportional to $G\left(1-p(t,a)\right)$. Given the rate at which new, successful infections occur Eq. **3**, the superinfection model (28) then yields the following expression for the rate of recovery from the infected status:

[5] $$R\left(\lambda_{s}\left(t\right),p\left(t,a\right)\right)\;=\;\frac{\lambda_{s}\left(t\right)}{\exp\left(c_{0}G\left(1-p(t,a)\right)\lambda_{s}\left(t\right)\right)-1}.$$

(see *SI Appendix* for the derivation).

Taken together, the infection dynamics of the host population is described by the following partial differential equations:

[6] $$\left\{\begin{array}{l} \frac{\partial s(t,a)}{\partial t}+\frac{\partial s(t,a)}{\partial a}=R(\lambda_{s}(t),p(t,a))\left(1-s(t,a)\right)-\lambda_{s}(t)s(t,a)\\ s(t,0)=1\\ \frac{\partial P(t,a)}{\partial t}+\frac{\partial P(t,a)}{\partial a}=G\left(1-p(t,a)\right)\lambda (t)-\delta P(t,a)\\ P(t,0)=0, \end{array}\right.$$

in which δ represents the turnover rate of genes in the parasite gene pool (see below). Note that even though all individuals with the same age have encountered the same number of pathogen genes P(t,a), the relationship between age and the number of pathogen genes encountered varies over time with changes in the force of infection. The number of pathogens genes encountered is therefore a truly independent variable characterizing the state of an individual and thus structuring the population.

### Dynamics of Parasite Diversity.

We assume that new parasite genes to emerge due to mitotic recombination inside infected hosts at a rate that is proportional to the total number of infections in the host population and the number of different gene pairs each parasite harbors. Following the superinfection model (28), the average number of infections in infected hosts of age a is given by:

$$E\left(\lambda_{s}\left(t\right),p\left(t,a\right)\right)=\frac{c_{0}G\left(1-p(t,a)\right)\lambda_{s}\left(t\right)}{1-\exp\left(-c_{0}G\left(1-p(t,a)\right)\lambda_{s}\left(t\right)\right)},$$

such that the rate of emergence of new parasite genes is given by:

[7] $$\begin{array}{l} \alpha \frac{G(G-1)}{2}E_{tot}(t)=\\ \;\;\;\;\;\;\;\;\;\;\;\;\;\alpha \frac{G(G-1)}{2}\int_{0}^{A_{m}}E\left(\lambda_{s}(t),p(t,a)\right)\frac{N\left(1-s(t,a)\right)\mu e^{-\mu a}}{1-\exp(-\mu A_{m})}da, \end{array}$$

with α a proportionality constant (for the recombination rate per gene per year). Emerging genes are furthermore assumed to establish with a probability derived from a classic result for the fixation probability of selected alleles in population genetics (49, 50)

[8] $$\Phi_{\mathrm{inv}}\left(t\right)=\frac{1-e^{-S(t)}}{1-e^{-E_{\mathrm{tot}}\left(t\right)S\left(t\right)}},$$

in which S(t) is the selection differential of a new gene (51), defined as:

[9] $$S\left(t\right)=\frac{\overset{¯}{p}(t)}{(1-\overset{¯}{p}(t))G},$$

where $\overset{¯}{p}(t)$ represents the average fraction of the parasite genes that hosts have encountered previously in their life:

[10] $$\overset{¯}{p}\left(t\right)=\int_{0}^{A_{m}}\frac{p\left(t,a\right)\mu e^{-\mu a}}{1-\exp(-\mu A_{m})}\;da.$$

The selection differential S(t) calculates the mean advantage of a parasite genome with a single novel gene out of a repertoire of G genes, relative to other parasite genomes from the existing pool.

Finally, we assume that genes disappear from the parasite gene pool at a rate δ and that immigration introduces new genes into the gene pool at a rate $P_{I}\lambda_{I}L$, in which $\lambda_{I}$ equals the immigration rate of infected hosts, L is the length of the parasite gene package and $P_{I}$ is an establishment probability of a new gene. Taken together, the dynamics of the parasite gene pool size D then follows the ordinary differential equation:

[11] $$\frac{\mathrm{dD}}{\mathrm{dt}}\;=\;\alpha \Phi_{\mathrm{inv}}\left(t\right)\frac{G(G-1)}{2}E_{\mathrm{tot}}\left(t\right)-\;\delta D\;+\;P_{I}\lambda_{I}L.$$

For the choice and estimates of parameter values, see *SI Appendix*.

### Computing Dynamics and Steady States.

Dynamics of the immune memory–structured SIS model specified by the partial differential equations [**6**] and the ordinary differential equation [**11**] were numerically simulated using the Escalator Boxcar Train method, a numerical method specifically designed for structured population models (52).

Steady states of the immune memory–structured SIS model Eqs. **6** and **11** are completely determined by the equilibrium force of infection $\tilde{\lambda }$ and the parasite antigenic diversity in equilibrium $\tilde{D}$. Given values for $\tilde{\lambda }$ and $\tilde{D}$, the differential equations in Eqs. **6** and **11** can be solved, albeit only numerically, which in turn allows for (numerical) evaluation of the age-dependent integrals occurring in the model (e.g., Eqs. **1** and **7**).

The two unknowns $\tilde{\lambda }$ and $\tilde{D}$ to be solved for satisfy the steady-state versions of equations Eq. **6** and **11**:

[12] $$\left\{\begin{array}{l} \lambda =\text{Λ}(A_{m})+\frac{\lambda_{I}}{N}\\ D=\frac{\alpha \frac{G(G-1)}{2}\left(1-\exp\left(-\frac{\text{Π}(A_{m})}{(1-\text{Π}(A_{m}))\tilde{G}}\right)\right)\text{Δ}(A_{m})}{\delta \left(1-\exp\left(-\text{Δ}(A_{m})\frac{\text{Π}(A_{m})}{(1-\text{Π}(A_{m}))\tilde{G}}\right)\right)}. \end{array}\right.$$

In these expressions, $\Lambda (A_{m})$, $\Pi (A_{m})$, and $\Delta (A_{m})$ are integral quantities, evaluated over the (age-dependent) life history of a host from its birth till its maximum lifespan, that represent the contribution of a host to the force of infection ($\Lambda (A_{m})$), the fraction of the parasite gene pool that a host encounters during its life ($\Pi (A_{m})$) and the total number of infections in the host population ($\Delta (A_{m})$). These integral life history quantities all represent expected, average values over the host population, taking into account its stable age distribution. To compute model steady states, we follow the approach introduced by Kirkilionis et al. (53) and evaluate these life history integrals by means of numerical integration of a system of ordinary differential equations (ODEs). Computing the model steady state for a given parameter set then involves the numerical solution of the nonlinear system of equations [**12**] using a Newton–Raphson iterative approach, whereby every evaluation of the right-hand side of this system of equations requires the numerical integration of a system of ODEs for $\Lambda (A_{m})$, $\Pi (A_{m})$, and $\Delta (A_{m})$ (see *SI Appendix* for details). Finally, to compute curves of steady-state solutions as a function of model parameter, we use the R package FindCurve (54), which implements a set of routines for curve continuation and the detection of bifurcation points.

## Supplementary Material

Appendix 01 (PDF)

## Data Availability

Previously published data were used for this work (29).
