# Supplementary material for: An immune memory–structured SIS epidemiological model for hyperdiverse pathogens
Source: Proc Natl Acad Sci U S A. 2023 Nov 1;120(45):e2218499120. doi: 10.1073/pnas.2218499120 (PMC10636369; doi:10.1073/pnas.2218499120)
Supplement: Supplementary file 1 — Appendix 01 (PDF) [file pnas.2218499120.sapp.pdf]

PNAS
www.pnas.org

Supplementary Information for

An immune memory-structured SIS epidemiological model for hyper-diverse pathogens

André M. de Roos, Qixin He and Mercedes Pascual

Mercedes Pascual
E-mail: pascualmm@uchicago.edu ; current E-mail: mercedes.pascual@nyu.edu

This PDF file includes:

Supplementary text
Figs. S1 to S6
Table S1
SI References

Supporting Information Text

Model formulation and analysis

Infection dynamics. We formulate an age-structured SI model in terms of susceptible, $S(t,a)$, and infected, $I(t,a)$, individuals as also studied in (1). The key features that set our model apart from other age-structured SI models, such as models with multiple classes of susceptible and infected individuals (1) or accounting for superinfection (2), are (i) that during their lifetime individuals build up a (variable) level of protection against infection depending on their infection history and (ii) that the infection dynamics and build-up of protection of host individuals not only lead to changes in the prevalence of the pathogen, but also to changes in pathogen diversity through the generation of new antigen-encoding genes. This interaction translates into a positive feedback mechanism, in which an increase in pathogen diversity leads to a decrease in built-up protection in hosts and consequently higher infection rates that increase pathogen diversity further. More generally, our model links dynamics within the infected host individual explicitly to changes in prevalence as well as characteristics of the pathogen.

Both susceptible and infected individuals are assumed to experience a constant mortality rate μ, but also to die instantaneously on reaching their maximum lifespan, equal to A_m. Susceptible and infected individuals produce (exclusively susceptible) offspring at a rate $\mu/\left(1-\exp\left(-\mu A_m\right)\right)$, which ensures that the total population size N is constant. The stable population age-distribution is hence given by:

$$n(a) \;=\; \frac{\mu}{1-\exp(-\mu A_m)} N\exp(-\mu a) \qquad \text{[S1]}$$

The constant population size allows the model to be expressed in terms of the fraction of susceptible individuals at age a:

$$s(t,a) \;=\; \frac{S(t,a)}{S(t,a)+I(t,a)} \;=\; \frac{S(t,a)}{n(a)}$$

Assuming that susceptible individuals get infected at a rate $\lambda(t)$, whereas infected individuals recover from infection at a rate $\tau(t)$, the dynamics of the fraction of susceptible individuals $s(t,a)$ is described by the following partial differential equation (PDE):

$$\begin{cases} \dfrac{\partial s(t,a)}{\partial t} + \dfrac{\partial s(t,a)}{\partial a} \;=\; \tau(t)\left(1-s(t,a)\right) \;-\; \lambda(t)\, s(t,a) \\ s(t,0) \;=\; 1 \end{cases} \qquad \text{[S2]}$$

We assume the infection process to be frequency- rather than density-dependent, reflecting that a random individual is drawn from the population (i.e. from the stable population age-distribution) as the donor and that infection may occur if this donor host is infected. We furthermore assume that migration of infected hosts from outside the population considered increases the force of infection by an amount λ_I/N, such that the force of infection $\lambda(t)$ is given by:

$$\lambda(t) \;=\; k_0 \frac{\mu}{1-\exp(-\mu A_m)} \int_0^{A_m} \left(1-s(t,a)\right)\, e^{-\mu a}\, da + \frac{\lambda_I}{N} \qquad \text{[S3]}$$

In the context of malaria, we focus on the antigenic diversity of the pathogen from a multigene family as the key determinant of its epidemiological parameters. More specifically, we assume that hosts receive infectious mosquito bites at a rate that is independent of their age and infection status and that every time an infectious bite takes place, a package of L genes encoding for different variant surface antigens is transferred. As a shorthand, these variants will be referred to hereafter as 'genes'. The L genes transferred are assumed to be randomly sampled from a pool of available genes in the pathogen population, the total size of which we indicate with D. The expected number of unique genes delivered in a biting event then follows a recurrence relation:

$$\mathbf{E}\left(Y_L|Y_{L-1}\right) = Y_{L-1} + \frac{D-Y_{L-1}}{D} \qquad \text{[S4]}$$

in which Y_{L-1} refers to the number of unique items chosen after $L-1$ picks from a pool that consists of D different items and

$$(D-Y_{L-1})/D \qquad \text{[S5]}$$

equals the probability to pick a new, unique item in the next pick. Given that $Y_0=0$ the expected number of different items after L picks $\mathbf{E}\left(Y_L\right)$ is given by:

$$\mathbf{E}\left(Y_L\right) = \begin{cases} 0 & \text{if } L=0 \\ 1 + \dfrac{(D-1)}{D}\mathbf{E}\left(Y_{L-1}\right) & \text{otherwise} \end{cases} \qquad \text{[S6]}$$

For every $L \geq 0$, the expected number G of unique genes delivered per infection event is then related to the package size L following:

$$G \;=\; \mathbf{E}(Y_L) = \frac{1-\left(\dfrac{D-1}{D}\right)^L}{1-\dfrac{D-1}{D}} = D\left(1-\left(\frac{D-1}{D}\right)^L\right)$$

We assume that the protection of a particular host against infection is related to its infection history, i.e. to the number of genes (or more specifically to the products of these genes) that it has been exposed to from previous infections. Let $P(t,a)$ indicate this cumulative number of genes that a host has encountered up to age a. As it is impossible to keep track precisely of which pathogen genes have already been encountered by a particular host, we assume complete homogenization with hosts building up a memory of the number, but not the identity of the genes encountered, while at every infectious biting event a host receives a package of G randomly drawn genes from the pathogen gene pool. As a host at age a has already encountered a fraction $p(t,a) = P(t,a)/D$ of the entire gene pool, the probability that a host has already encountered all the genes that are transferred in the infectious bite can be approximated by $p(t,a)^G$. If a host has previously encountered all the genes, it is assumed that the infection is unsuccessful. The probability of actually becoming infected following an infectious bite therefore equals $1 - p(t,a)^G$, such that the rate at which successful infections occur is given by:

$$\left(1 - p(t,a)^G\right)\lambda(t) \tag{S7}$$

The dynamics of $P(t,a)$ can be derived by considering the expected number of new genes delivered in a biting event. The number of new genes delivered follows a binomial distribution with probability $1 - p(t,a)$, such that the expected number of new genes delivered is given by:

$$\sum_{i=0}^{i=G} i \binom{G}{i} \left(1 - p(t,a)\right)^i p(t,a)^{(G-i)}$$

This mean number of genes delivered has to be conditioned, however, on the probability that a biting event leads to a successful infection:

$$\frac{\sum_{i=0}^{i=G} i \binom{G}{i} \left(1 - p(t,a)\right)^i p(t,a)^{(G-i)}}{1 - p(t,a)^G} = \frac{G\left(1 - p(t,a)\right)}{1 - p(t,a)^G}$$

Given that the rate at which successful infections occur equals $\left(1 - p(t,a)^G\right)\lambda(t)$, the rate of delivery of new genes equals:

$$G\left(1 - p(t,a)\right)\lambda(t)$$

The dynamics of $P(t,a)$ is hence described by the following PDE:

$$\frac{\partial P(t,a)}{\partial a} + \frac{\partial P(t,a)}{\partial a} = G\left(1 - p(t,a)\right)\lambda(t) - \delta P(t,a) \tag{S8}$$

In this equation δ represents a rate at which genes are lost from the pool so that the memory of these genes is no longer relevant. We will discuss this loss in more detail when modelling the dynamic changes in the diversity D of the gene pool (see below).

We assume that all host individuals accumulate new pathogen types at the same rate, irrespective of their infection status, that is, irrespective of whether they are susceptible or infected. Infected individuals can therefore become super-infected. We adopt a superinfection model (3) to describe the recovery rate from the infected into the susceptible class. Dietz et al. (3) provide the following equation for the rate of recovery of infected individuals:

$$R(h) = \frac{h}{\exp(h/r) - 1}$$

in which h equals the force of infection, that is the rate at which new, successful infections occur, while r equals the rate at which a single infection is cleared. According to this model the equilibrium number of inoculations present at any time is a Poisson random variable with mean h/r. As argued above, the rate at which new, successful infections occur is given by:

$$h = \left(1 - p(t,a)^G\right)\lambda(t)$$

The duration of each single infection will be assumed to decrease with the number of genes that the host has already encountered before and therefore be proportional to $G\left(1 - p(t,a)\right)$ with proportionality constant c_0 (the duration of infection corresponding to the expression of a single gene). Hence, the rate of recovery from an infection equals:

$$r = \frac{1}{c_0 G\left(1 - p(t,a)\right)}$$

yielding the following expression for the rate of recovery from the infected status:

$$\tau(t) = R(\lambda(t), p(t,a)) = \frac{\left(1 - p(t,a)^G\right)\lambda(t)}{\exp\left(c_0 G\left(1 - p(t,a)\right)\left(1 - p(t,a)^G\right)\lambda(t)\right) - 1} \tag{S9}$$

Taken together this leads to the following system of equations describing the infection dynamics in our model:

$$\begin{cases} \dfrac{\partial s(t,a)}{\partial t} + \dfrac{\partial s(t,a)}{\partial a} = R(\lambda(t), p(t,a))\,(1 - s(t,a)) - \left(1 - p(t,a)^G\right)\lambda(t)s(t,a) \\ s(t,0) \;=\; 1 \\ \dfrac{\partial P(t,a)}{\partial t} + \dfrac{\partial P(t,a)}{\partial a} \;=\; G\,(1 - p(t,a))\,\lambda(t) \;-\; \delta P(t,a) \\ P(t,0) \;=\; 0 \\ \lambda(t) \;=\; k_0 \dfrac{\mu}{1 - \exp(-\mu A_m)} \displaystyle\int_0^{A_m} (1 - s(t,a))\, e^{-\mu a}\, da \;+\; \dfrac{\lambda_I}{N} \\ p(t,a) = \dfrac{P(t,a)}{D} \\ G = D\left(1 - \left(\dfrac{D-1}{D}\right)^L\right) \\ R(\lambda, p) \;=\; \dfrac{\left(1 - p^G\right)\lambda}{\exp\left(c_0 G\,(1-p)\left(1 - p^G\right)\lambda\right) - 1} \end{cases} \qquad \text{[S10]}$$

Note that the state of an individual in our model is determined by its age a and the number of pathogen genes P it has encountered since its birth. The relationship between individual age and the number of pathogen genes encountered is however not constant, but varies dynamically with the individual's history of exposure, when the force of infection changes over time. The quantity P is therefore an independent variable characterizing the state of an individual. This is similar to models where individuals are classified as a function of age and size, whereby one could express the size of an individual as a (time-varying) function of its age. Technically, the individual state space is hence 2-dimensional and we could have written the model in terms of a PDE for a density function $s(t,a,P)$ over the state space spanned by the individual age and the number of pathogens the individual has encountered. However, the unique state at birth ($a = 0, P = 0$) implies that the support of this density function is only 1-dimensional, but that this support varies dynamically with the force of infection and the history of exposure. Because of this (dynamically changing) one-dimensional support it is more appropriate to formulate the model in terms of two separate PDEs, one for $P(t,a)$ (capturing the dynamics of the 1-dimensional support) and one for $s(t,a)$ (the fraction of susceptibles along the 1-dimensional support), rather than a single PDE for $s(t,a,P)$ (see ref. (4), for more details).

Diversity dynamics. We assume that pathogen diversity increases as a consequence of the recombination of pathogen genes within infected hosts. More specifically, we assume that the diversity of the antigen-encoding genes increases at a rate proportional to the total number of infections, $E_{tot}(t)$, in the host population as well as to the number of different gene pairs that each parasite harbors. Given that a parasite harbors G different genes, the expected number of different gene pairs equals $G(G-1)/2$. Following the superinfection model of Dietz et al. (3) the average number of infections in infected hosts of age a, indicated with $E(\lambda(t), p(t,a))$, is given by:

$$E(\lambda(t), p(t,a)) \;=\; \frac{h/r}{1 - \exp(-h/r)} \;=\; \frac{c_0 G\,(1 - p(t,a))\left(1 - p(t,a)^G\right)\lambda(t)}{1 - \exp\left(-c_0 G\,(1 - p(t,a))\left(1 - p(t,a)^G\right)\lambda(t)\right)}$$

The total number of infections in the entire population (counting both single and multiple infections) is hence given by the integral:

$$E_{tot}(t) = \frac{\mu N}{1 - \exp(-\mu A_m)} \int_0^{A_m} E\left(\lambda(t), p(t,a)\right)(1 - s(t,a))\, e^{-\mu a}\, da$$

The rate at which new genes emerge is hence given by:

$$\alpha \frac{G(G-1)}{2} E_{tot}(t) = \frac{G(G-1)}{2} \frac{\mu N}{1 - \exp(-\mu A_m)} \int_0^{A_m} E\left(\lambda(t), p(t,a)\right)(1 - s(t,a))\, e^{-\mu a}\, da$$

where α represents the recombination rate per gene per year per infection.

A new gene will, however, only get established in the parasite gene pool if it survives the initial stochastic phase when its frequency is still low, the probability of which is determined by its selection coefficient and the total number of infections:

$$\Phi_{inv}(t) = \frac{1 - e^{-S(t)}}{1 - e^{-E_{tot}(t)S(t)}}$$

in which $S(t)$ is the selection differential of a new gene, defined as:

$$S(t) = \frac{W_{new}}{\bar{W}} - 1 = \frac{(1-\bar{p}(t))(G-1)+1}{(1-\bar{p}(t))G} - 1 = \frac{\bar{p}(t)}{(1-\bar{p}(t))G}$$

In the expression for $S(t)$, W_{new} represents the fitness of a parasite genome containing this novel gene, while $\bar{W}$ is the average fitness of parasite genomes composed of existing genes, which is determined by $\bar{p}(t)$, the average fraction of the genes that has been seen by the host population:

$$\bar{p}(t) = \frac{\mu}{1-\exp(-\mu A_m)} \int_0^{A_m} p(t,a)\, e^{-\mu a}\, da$$

Thus, the selection differential $S(t)$ calculates the mean advantage of a parasite genome with a single, novel gene out of the package of G genes compared to other parasite genomes with G genes from the existing pool (5).

The rate at which new genes are generated and become frequent in the entire population (counting both single and multiple infections) is hence given by:

$$\alpha \Phi_{inv}(t) \frac{G(G-1)}{2} E_{tot}(t) = \alpha \frac{G(G-1)}{2} \Phi_{inv}(t) \frac{\mu N}{1-\exp(-\mu A_m)} \int_0^{A_m} E\left(\lambda(t), p(t,a)\right)(1-s(t,a))\; e^{-\mu a}\, da$$

Parasite diversity can also increase through immigration of infected individuals, which occurs at a rate λ_I (see Eq. (S3)), if these individuals carry new genes. We assume that these immigrating, infected hosts introduce new genes into the gene pool at a rate $P_I L \lambda_I$, where the parameter P_I accounts for the probability that a gene of the immigrating individual is not part of the gene pool already as well as the probability that this new gene gets established in the gene pool. Finally, we assume genes to disappear from the gene pool at a constant turn-over rate δ due to stochastic loss. This turn-over rate also occurs in the PDE (S8) as the number of genes out of the current gene pool seen by an individual of age a at time t also is prone to this turn-over rate.

Summarizing, the dynamics of the parasite diversity D is given by the following system of equations:

$$\begin{cases} E(\lambda, p) \;=\; \dfrac{c_0 G\,(1-p(t,a))\left(1-p(t,a)^G\right)\lambda(t)}{1-\exp\left(-c_0 G\,(1-p(t,a))\left(1-p(t,a)^G\right)\lambda(t)\right)} \\[2ex] E_{tot}(t) = \dfrac{\mu N}{1-\exp(-\mu A_m)} \displaystyle\int_0^{A_m} E\left(\lambda(t), p(t,a)\right)(1-s(t,a))\; e^{-\mu a}\, da \\[2ex] \bar{p}(t) = \dfrac{\mu}{1-\exp(-\mu A_m)} \displaystyle\int_0^{A_m} p(t,a)\, e^{-\mu a}\, da \\[2ex] S(t) = \dfrac{\bar{p}(t)}{(1-\bar{p}(t))G} \\[2ex] \Phi_{inv}(t) = \dfrac{1-e^{-S(t)}}{1-e^{-E_{tot}(t)S(t)}} \\[2ex] \dfrac{dD}{dt} \;=\; \alpha \Phi_{inv}(t) \dfrac{G(G-1)}{2} E_{tot}(t) - \;\delta D \;+\; P_I \lambda_I L \end{cases} \qquad \text{[S11]}$$

The complete model for the interplay and feedback between the epidemiological spread of the parasite in the host population and the diversity of the pathogen itself is therefore given by the two systems of equations (S10) and (S11).

Computing equilibrium states. Let $\tilde{s}(a)$, $\tilde{P}(a)$, $\tilde{\lambda}$ and $\tilde{D}$ refer to the equilibrium values of the dynamic variables $s(t,a)$, $P(t,a)$, $\lambda(t)$ and $D(t)$, respectively. Computing equilibrium states of the model described by the two systems of equations (S10) and (S11) boils down to solving the values of $\tilde{\lambda}$ and $\tilde{D}$ from the following set of equations:

$$\tilde{\lambda} = k_0 \frac{\mu}{1-\exp(-\mu A_m)} \int_0^{A_m} (1-\tilde{s}(a))\; e^{-\mu a}\, da \;+\; \frac{\lambda_I}{N}$$

$$\tilde{D} = \frac{\alpha \dfrac{G(G-1)}{2} \tilde{\Phi}_{inv} \tilde{E}_{tot} + P_I \lambda_I L}{\delta}$$

in which $\tilde{\Phi}_{inv}$ and $\tilde{E}_{tot}$ refer to the equilibrium values of $\Phi(t)$ and $E_{tot}(t)$, respectively, that are given by:

$$\tilde{\Phi}_{inv} = \frac{1-\exp\left(-\tilde{S}\right)}{1-\exp\left(-\tilde{E}_{tot}\tilde{S}\right)}$$

$$\tilde{E}_{tot} = \frac{\mu N}{1-\exp(-\mu A_m)} \int_0^{A_m} E\left(\tilde{\lambda}, \tilde{p}(a)\right)(1-\tilde{s}(a))\; e^{-\mu a}\, da$$

with $E(\tilde{\lambda}, \tilde{p}(a))$ and $\tilde{S}$ being equal to:

$$E(\tilde{\lambda}, \tilde{p}(a)) = \frac{c_0 \tilde{G}\,(1 - \tilde{p}(a))\left(1 - \tilde{p}(a)^{\tilde{G}}\right)\tilde{\lambda}}{1 - \exp\left(-c_0 \tilde{G}\,(1 - \tilde{p}(a))\left(1 - \tilde{p}(a)^{\tilde{G}}\right)\tilde{\lambda}\right)}$$

$$\tilde{S} = \frac{\dfrac{\mu}{1 - \exp(-\mu A_m)} \displaystyle\int_0^{A_m} \tilde{p}(a)\, e^{-\mu a}\, da}{\left(1 - \dfrac{\mu}{1 - \exp(-\mu A_m)} \displaystyle\int_0^{A_m} \tilde{p}(a)\, e^{-\mu a}\, da\right)\tilde{G}}$$

and $\tilde{G}$ representing the equilibrium value of G, which is related to $\tilde{D}$ by:

$$\tilde{G} \;=\; \tilde{D}\left(1 - \left(\frac{\tilde{D} - 1}{\tilde{D}}\right)^L\right)$$

Given a constant parasite diversity $\tilde{D}$ and force of infection $\tilde{\lambda}$, the cumulative number of genes encountered by hosts of age a in an equilibrium state can then be derived by solving the PDE (S8), resulting in:

$$\tilde{P}(a) \;=\; \frac{\tilde{G}\tilde{\lambda}\tilde{D}}{\tilde{G}\tilde{\lambda} + \delta\tilde{D}}\left(1 \;-\; \exp\left(-\left(\frac{\tilde{G}\tilde{\lambda}}{\tilde{D}} + \delta\right) a\right)\right)$$

which results in an explicit expression for the fraction of the genes encountered by a host up to age a:

$$\tilde{p}(a) \;=\; \frac{\tilde{G}\tilde{\lambda}}{\tilde{G}\tilde{\lambda} + \delta\tilde{D}}\left(1 \;-\; \exp\left(-\left(\frac{\tilde{G}\tilde{\lambda}}{\tilde{D}} + \delta\right) a\right)\right)$$

Such an explicit expression can not be derived for the fraction of susceptible hosts of age a, $\tilde{s}(a)$, which can therefore only be computed by numerically integrating the ordinary differential equation (ODE):

$$\frac{d\tilde{s}(a)}{da} \;=\; R(\tilde{\lambda}, \tilde{p}(a))\,(1 - \tilde{s}(a)) \;-\; \left(1 - \tilde{p}(a)^{\tilde{G}}\right)\tilde{\lambda}\tilde{s}(a), \qquad \text{with } \tilde{s}(0) \;=\; 1$$

in which $R(\tilde{\lambda}, \tilde{p}(a))$ is given by:

$$R(\tilde{\lambda}, \tilde{p}(a)) \;=\; \frac{\left(1 - \tilde{p}(a)^{\tilde{G}}\right)\tilde{\lambda}}{\exp\left(c_0 \tilde{G}\,(1 - \tilde{p}(a))\left(1 - \tilde{p}(a)^{\tilde{G}}\right)\tilde{\lambda}\right) - 1}$$

To compute the integrals in the preceding conditions determining the steady state of the immune memory-structured SI-model we follow the approach introduced by Kirkilionis et al. (6) to numerically evaluate these integrals by means of numerical integration of a system of ODEs. To that end, define the following age-dependent quantities.

$$\Lambda(a) = k_0 \frac{\mu}{1 - \exp(-\mu A_m)} \int_0^a (1 - \tilde{s}(\xi))\; e^{-\mu\xi}\, d\xi$$

$$\Delta(a) = \frac{\mu N}{1 - \exp(-\mu A_m)} \int_0^a E\left(\tilde{\lambda}, \tilde{p}(\xi)\right)(1 - \tilde{s}(\xi))\; e^{-\mu\xi}\, d\xi$$

$$\Pi(a) = \frac{\mu}{1 - \exp(-\mu A_m)} \int_0^a \tilde{p}(\xi)\, e^{-\mu\xi}\, d\xi$$

Differentiating the right-hand sides of these expressions with respect to a results in the following system of ODEs:

$$\frac{d\Lambda}{da} = k_0 \frac{\mu}{1 - \exp(-\mu A_m)} (1 - \tilde{s}(a))\; e^{-\mu a}$$

$$\frac{d\Delta}{da} = \frac{\mu N}{1 - \exp(-\mu A_m)} E\left(\tilde{\lambda}, \tilde{p}(a)\right)(1 - \tilde{s}(a))\; e^{-\mu a}$$

$$\frac{d\Pi}{da} = \frac{\mu}{1 - \exp(-\mu A_m)} \tilde{p}(a)\, e^{-\mu a}$$

with initial conditions $\Lambda(0) = \Delta(0) = \Pi(0) = 0$.

Summarizing, the steady-state of the immune memory-structured SI model defined by the two systems of equations (S10) and (S11) is determined by the conditions:

$$\begin{cases} \lambda \ = \ \Lambda(A_m) \ + \ \dfrac{\lambda_I}{N} \\ D = \dfrac{\alpha \dfrac{G(G-1)}{2}\left(1 - \exp\left(-\dfrac{\Pi(A_m)}{(1-\Pi(A_m))\tilde{G}}\right)\right)\Delta(A_m)}{\delta\left(1 - \exp\left(-\Delta(A_m)\dfrac{\Pi(A_m)}{(1-\Pi(A_m))\tilde{G}}\right)\right)} \end{cases} \tag{S12}$$

This system of non-linear equations has to be solved iteratively using a Newton method, whereby each computation of the right-hand side of this condition requires numerical integration of the following system of ODEs:

$$\begin{cases} \dfrac{d\tilde{s}}{da} \ = \ R(\tilde{\lambda}, \tilde{p}(a))\,(1 - \tilde{s}(a)) \ - \ \left(1 - \tilde{p}(a)^{\tilde{G}}\right)\tilde{\lambda}\tilde{s}(a) & \tilde{s}(0) \ = \ 1 \\ \dfrac{d\Lambda}{da} \ = \ k_0 \dfrac{\mu}{1 - \exp(-\mu A_m)}\,(1 - \tilde{s}(a))\, e^{-\mu a} & \Lambda(0) \ = \ 0 \\ \dfrac{d\Delta}{da} \ = \ \dfrac{\mu N}{1 - \exp(-\mu A_m)} E(\tilde{\lambda}, \tilde{p}(a))\,(1 - \tilde{s}(a))\, e^{-\mu a} & \Delta(0) \ = \ 0 \\ \dfrac{d\Pi}{da} \ = \ \dfrac{\mu}{1 - \exp(-\mu A_m)} \tilde{p}(a) e^{-\mu a} & \Pi(0) \ = \ 0 \end{cases} \tag{S13}$$

in which $\tilde{G}$, $\tilde{p}(a)$, $R(\tilde{\lambda}, \tilde{p}(a))$ and $E(\tilde{\lambda}, \tilde{p}(a))$ are defined as:

$$\begin{cases} \tilde{G} & = \ \tilde{D}\left(1 - \left(\dfrac{\tilde{D}-1}{\tilde{D}}\right)^L\right) \\ \tilde{p}(a) & = \ \dfrac{\tilde{G}\tilde{\lambda}}{\tilde{G}\tilde{\lambda} + \delta\tilde{D}}\left(1 \ - \ \exp\left(-\left(\dfrac{\tilde{G}\tilde{\lambda}}{\tilde{D}} + \delta\right) a\right)\right) \\ R(\tilde{\lambda}, \tilde{p}(a)) & = \ \dfrac{\left(1 - \tilde{p}(a)^{\tilde{G}}\right)\tilde{\lambda}}{\exp\left(c_0 \tilde{G}\,(1 - \tilde{p}(a))\left(1 - \tilde{p}(a)^{\tilde{G}}\right)\tilde{\lambda}\right) - 1} \\ E(\tilde{\lambda}, \tilde{p}(a)) & = \ \dfrac{c_0 \tilde{G}\,(1 - \tilde{p}(a))\left(1 - \tilde{p}(a)^{\tilde{G}}\right)\tilde{\lambda}}{1 - \exp\left(-c_0 \tilde{G}\,(1 - \tilde{p}(a))\left(1 - \tilde{p}(a)^{\tilde{G}}\right)\tilde{\lambda}\right)} \end{cases} \tag{S14}$$

Disease invasion into closed populations. Our model represents an open system in which there is a contribution to the force of infection from outside the system through immigration, which we consider the most realistic setup for local malaria dynamics. Because of this immigration the diversity never drops below a minimum level even at very low transmission intensity, which in turn allowed us to make the simplifying assumption of a constant loss rate of pathogen genes. In the absence of any immigration, however, the constant loss rate of diversity would imply that diversity approaches 0, which is biologically unrealistic as on invasion into a disease-free population the pathogen would be characterized by a given value of distinct antigenic-encoding genes (which should at least be one, and could be up to L). To explore the invasion of the pathogen into a disease-free population that is closed to any immigration, we therefore would have to reformulate the model to ensure that the diversity of pathogen genes never drops below some threshold value, which can be achieved by replacing the constant, per-gene loss rate of diversity δ with the diversity-dependent loss rate:

$$\delta \exp\left(1 - \frac{D}{D_{min}}\right) \tag{S15}$$

in which the parameter $D_{min} > 1$ is a minimum value of diversity. The differential equation describing the dynamics of the parasite diversity D would in this case be

$$\frac{dD}{dt} \ = \ \alpha\Phi_{inv}(t)\frac{G(G-1)}{2}E_{tot}(t) - \ \delta D \exp\left(1 - \frac{D}{D_{min}}\right) \tag{S16}$$

where we have dropped the immigration term as we assume $\lambda_I = 0$.

This modified model exhibits the classical pattern of a stable, disease-free equilibrium at low transmission intensity (low values of the contact rate k_0), exchanging stability with an endemic equilibrium with low diversity and prevalence when transmission intensity increases, which is the standard scenario of disease invasion typically associated with studies of R_0 (see Fig S5 and S6). Importantly, though, the bistability and the associated saddle-node bifurcation that occurs when transmission intensity is sufficiently high, is unaffected by this change in model structure. Figure S6 shows that the exact position of the transcritical bifurcation where invasion of the pathogen into the disease-free equilibrium becomes possible (i.e. where $R_0 = 1$) depends on the minimum value D_{min}.

A simplified, ordinary differential equation model

If we assume that $p(t,a)$, the fraction of diversity encountered by an individual of age a, is constant, the model equations (S10) and (S11) can be significantly simplified. More specifically, let's assume that

$$p(t,a) = p$$

with p a parameter value. As a consequence also $\bar{p}(t)$ is constant and equal to p. The PDE for $P(t,a)$ in equations (S10) can be dropped entirely. Furthermore, assume that there is no limit to the lifespan of host individuals such that $A_m = \infty$ and define

$$I(t) = \int_0^\infty (1 - s(t,a))\, \mu e^{-\mu a}\, da$$

as the fraction of infected individuals in the entire population. The integrals in the expressions for $\lambda(t)$ and $E_{tot}(t)$ in equations (S10) can then be written in terms of $I(t)$, while the PDE for $s(t,a)$ can be rewritten as an ODE for $I(t)$:

$$\begin{aligned}
\frac{dI}{dt} &= \frac{d}{dt}\int_0^\infty (1 - s(t,a))\, \mu e^{-\mu a}\, da \\
&= \int_0^\infty \frac{\partial s(t,a)}{\partial a} \mu e^{-\mu a}\, da - R(\lambda(t)) \int_0^\infty (1 - s(t,a))\, \mu e^{-\mu a}\, da + \left(1 - p^G\right) \lambda(t) \int_0^\infty s(t,a) \mu e^{-\mu a}\, da \\
&= s(t,a)\mu e^{-\mu a}\Bigg|_{a=0}^{a=\infty} - \int_0^\infty -\mu^2 s(t,a) e^{-\mu a}\, da - R(\lambda(t)) I(t) + \left(1 - p^G\right) \lambda(t)\,(1 - I(t)) \\
&= -\mu s(t,0) + \mu \int_0^\infty s(t,a)\mu e^{-\mu a}\, da - R(\lambda(t)) I(t) + \left(1 - p^G\right) \lambda(t)\,(1 - I(t)) \\
&= \left(1 - p^G\right) \lambda(t)\,(1 - I(t)) - R(\lambda(t)) I(t) - \mu I(t)
\end{aligned}$$

The unstructured model that is analogous to the model in equations (S10) is hence given by the following equations:

$$\begin{cases}
\lambda(t) \;=\; k_0 I(t) \;+\; \dfrac{\lambda_I}{N} \\
G(t) = D(t)\left(1 - \left(\dfrac{D(t)-1}{D(t)}\right)^L\right) \\
R(\lambda) \;=\; \dfrac{\left(1 - p^{G(t)}\right)\lambda}{\exp\left(c_0 G(t)\,(1-p)\,(1 - p^{G(t)})\,\lambda\right) - 1} \\
E_{tot}(t) = \dfrac{c_0 G(t)\,(1-p)\left(1 - p^{G(t)}\right)\lambda}{1 - \exp\left(-c_0 G(t)\,(1-p)\,(1 - p^{G(t)})\,\lambda\right)} I(t) N \\
S(t) = \dfrac{p}{(1-p)G(t)} \\
\Phi_{inv}(t) = \dfrac{1 - e^{-S(t)}}{1 - e^{-E_{tot}(t)S(t)}} \\
\dfrac{dI}{dt} = \left(1 - p^{G(t)}\right)\lambda(t)\,(1 - I(t)) - R(\lambda(t)) I(t) - \mu I(t) \\
\dfrac{dD}{dt} \;=\; (\alpha \dfrac{G(t)(G(t)-1)}{2} + mutation)\Phi_{inv}(t) E_{tot}(t) -\; \delta D(t) \;+\; P_I \lambda_I L
\end{cases} \qquad \text{[S17]}$$

a

where $s(t,0) = 1$ has been used in the derivation of the ODE for $I(t)$ (since no individuals are born infectious). As default parameter values the same parameter values are used as for the immune memory-structured model. The only additional parameter in the ODE model is p, the fraction of the diversity that individuals have encountered already and are hence immune to.

We used the R package "deBif" (7) to numerically compute equilibrium curves of the simplified malaria model in terms of ordinary differential equations as function of model parameters and to compute the regions in parameter space where alternative stable states occur (see Figure S3).

Choice of parameter values

The range of values of the contact rate k_0 in the bifurcation analysis was chosen to encompass values of the force of infection consistent with those reported in the literature for fitted malaria models (1) and for measurements of the entomological inoculation rate, EIR, from high to low transmission regions.

The value of c_0 was estimated to be consistent with a duration of infection of about 1 year and the number of *var* genes in a single infection, given their sequential expression.

The rate of increase in diversity due to immigration is given in our model by $P_I \lambda_I L$. We adopted the default values $\lambda_I = 1000$ and $P_I = 5 \times 10^{-5}$ so that if the force of infection is of the order 1, immigration would be responsible for about 10% of the infections, at the default host population size of $N = 10000$. We varied the probability P_I above and below that value.

The mitotic recombination rate per gene within a parasite, α, was estimated from the in vitro experiments in (8)

The death rate of an average gene in the gene pool (or the inverse of lifespan of a gene), δ, was estimated by adapting a set of equations derived from population genetics in a system of negative frequency-dependent selection (NFDS) (9). In population genetics, processes are usually measured in units of the product of population size and generation time so that they can be more easily generalized. In transmission dynamics, the generation time of individual parasites can be approximated by $(1/k_0)/2$, because approximately one transmission event and one death event occur for a parasite during the period of $1/k_0$, resulting in twice of the variance compared to a standard Wright-Fisher model. Thus, recombination events in the system per generation occur at the rate of

$$M(t) = \alpha \Phi_{inv}(t) \frac{G(G-1)}{2} E_{tot}(t) \frac{2}{k_0}$$

NFDS per generation in units of total infections is given by the selective advantage of a new gene (Eq. 4 in (5)) times the number of total infections,

$$B(p,t) = S(t) E_{tot}(t) = \frac{p}{(1-p)G(t)} E_{tot}(t)$$

We further denote the mean frequency of a gene under balancing selection (from NFDS) as $f(t)$. At equilibrium, the evolution of genes should be at the mutation-selection-drift balance, which satisfies the following equation (see Eq. 4 in (9)):

$$2M(t) \exp(B(p,t) f(t)) \sqrt{\frac{\pi f(t)}{B(p,t)}} = 1$$

An expression for $f(t)$ can be solved using the above equation given a fixed fraction p of genes already seen by the hosts. Using a diffusion approximation, we can then compute the average lifespan of a gene in years given a starting frequency of $f(t)$ (modified from Eq. 6 in (9)):

$$T(f(t),p) = \frac{\sqrt{2}}{2M(t)B(p,t)f(t)} E_{tot}(t) \frac{2}{k_0}$$

Assuming an E_{tot} of 10,000, k_0 varying from 60 to 200, p from 0.1 to 0.9, and a value of α of $6.8 \cdot 10^{-5}$, the lifespan of a gene is roughly of the order of 10 years (7-17 years). On this basis, we chose a fixed δ value of 0.1 in the model.

Finally, we considered a length $L = 20$. This value was meant to represent the order of magnitude of the number of *var* genes in the genome of *P. falciparum* while allowing for some of the genes not being expressed and retained in immune memory.

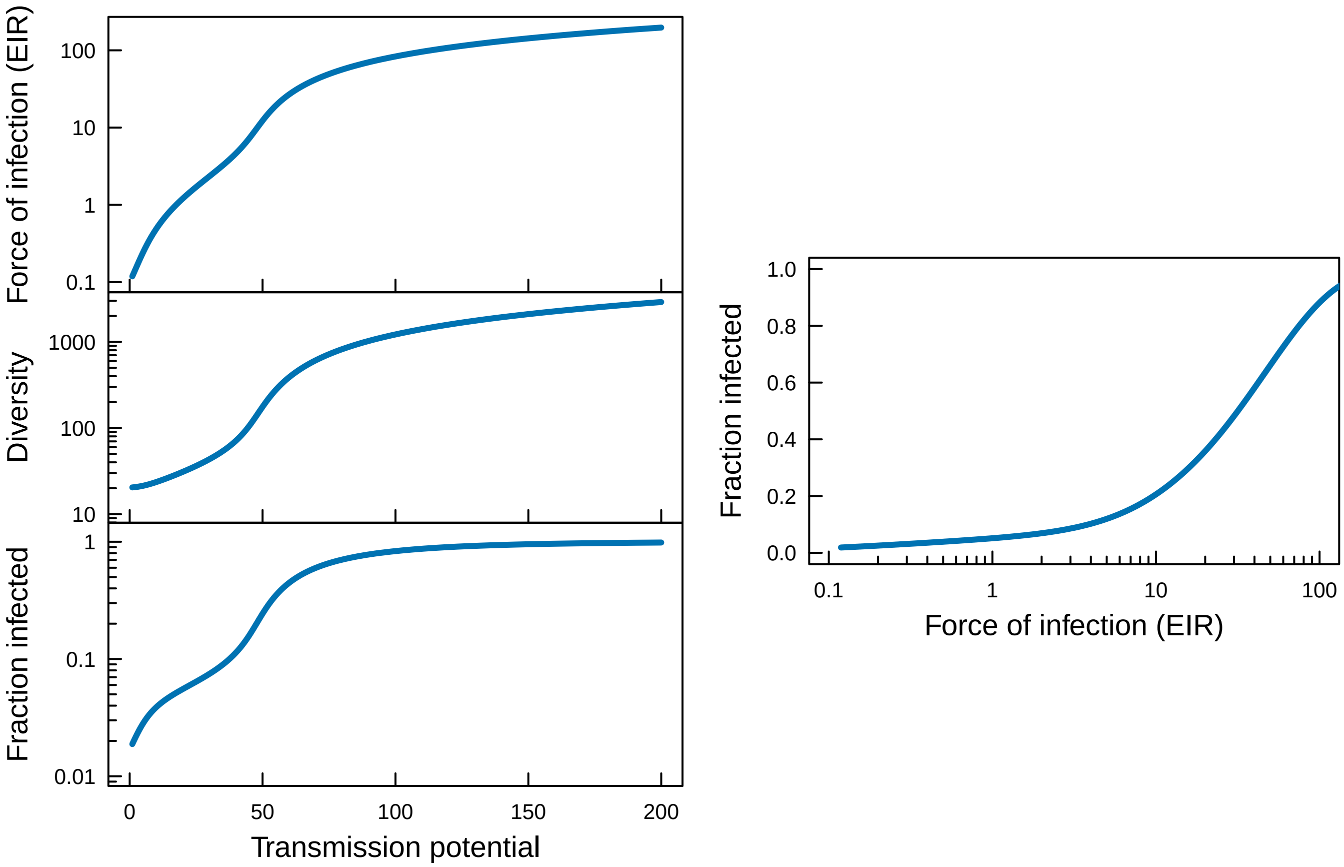

Fig. S1. Equilibrium states of the malaria model as a function of the transmission potential (*left*) and the relationship between the fraction of infected individuals in the population and the force of infection in the stable equilibrium states observed for different values of the transmission potential when the probability that immigration of infected hosts leads to an increase in the parasite diversity is twice its default value ($P_I = 1.0 \cdot 10^{-4}$ instead of $P_I = 5.0 \cdot 10^{-5}$.

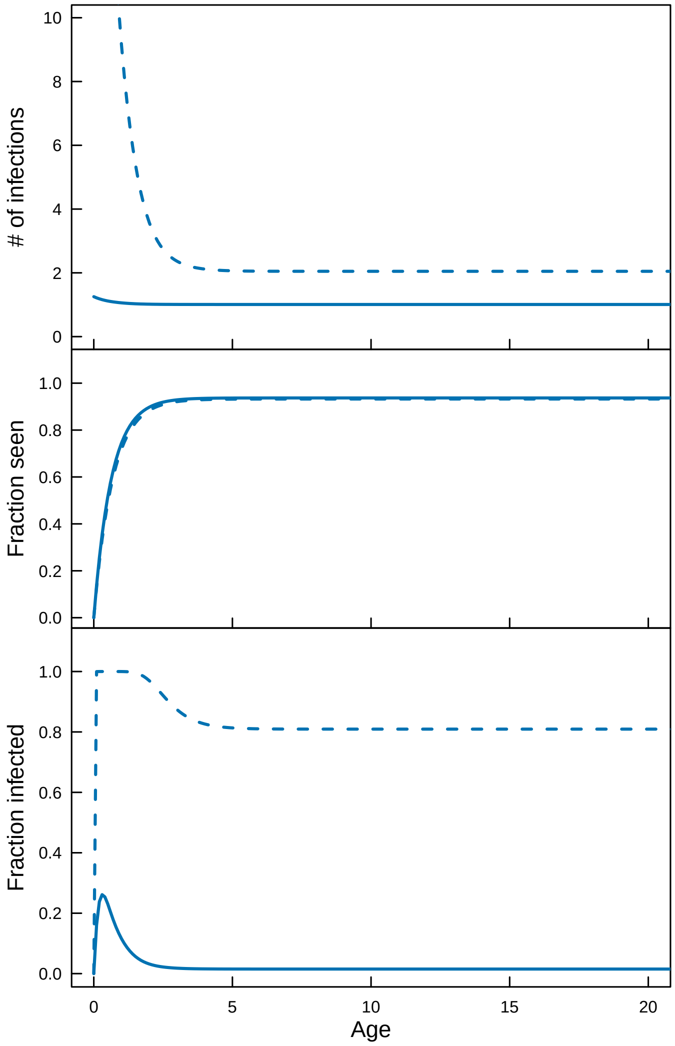

Fig. S2. Age-dependent development of the number of infections (*top*) that individuals carry, the fraction of the total parasite gene pool they have previously encountered (*middle*) and the fraction of individuals that are infected at any given time (*bottom*) in the low- (*solid lines*) and high-prevalence equilibrium (*dashed line*) at a transmission potential equal to $k_0 = 100$ (cf. Figure 1 in the main text).

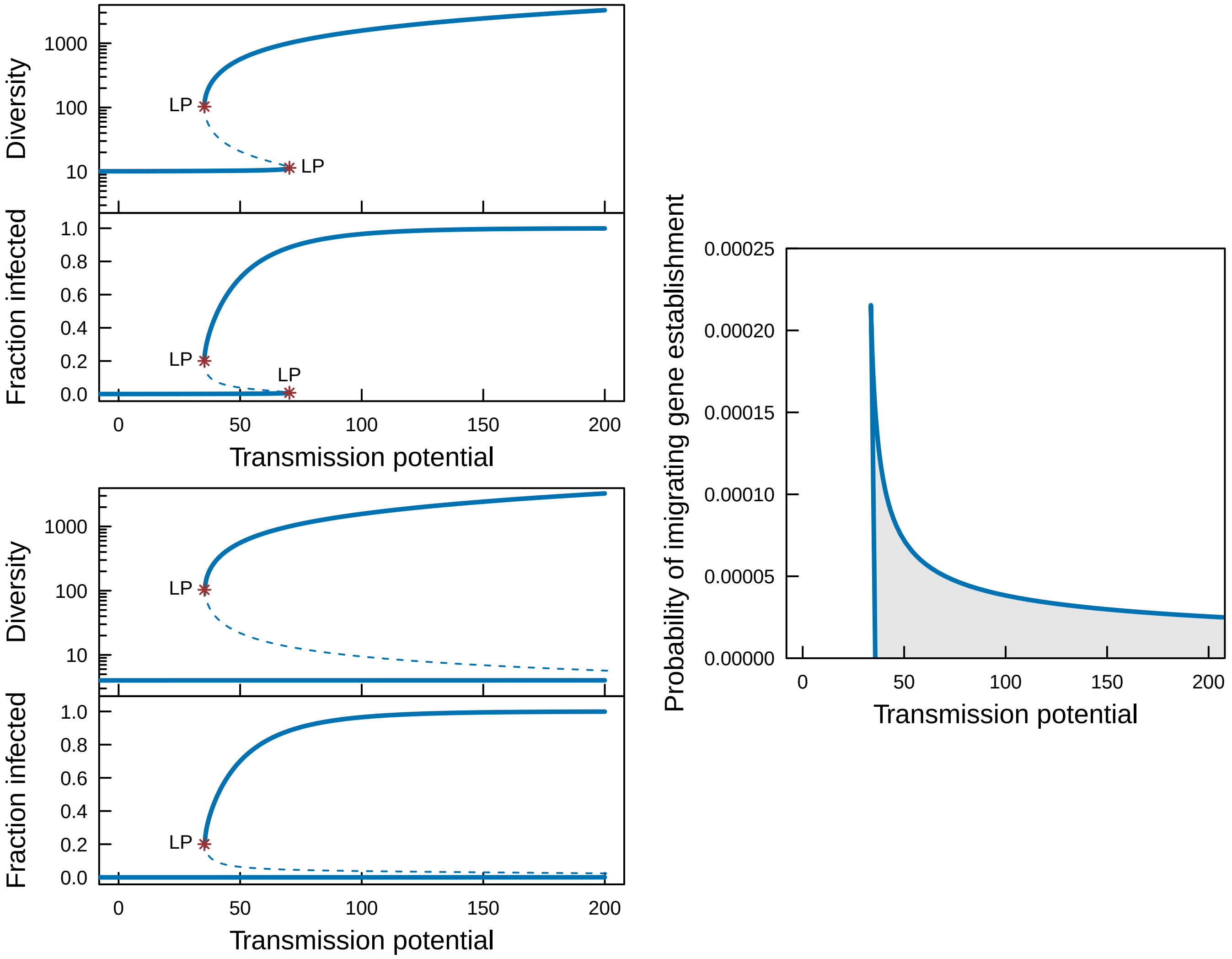

Fig. S3. Equilibrium states of the simplified malaria model in terms of ordinary differential equations Eq. (S17) as a function of the transmission potential for $P_I = 5 \cdot 10^{-5}$ (*top-left*) and for $P_I = 2 \cdot 10^{-5}$ (*bottom-left*). *Right*: Parameter domain (*grey*) for which alternative stable equilibrium states occur in the simplified malaria model in terms of ordinary differential equations Eq. (S17) as a function of the probability that an immigration event leads to the introduction of a new parasite gene (parameterized in the model by P_I) and the transmission potential, the contact rate parameter k_0.

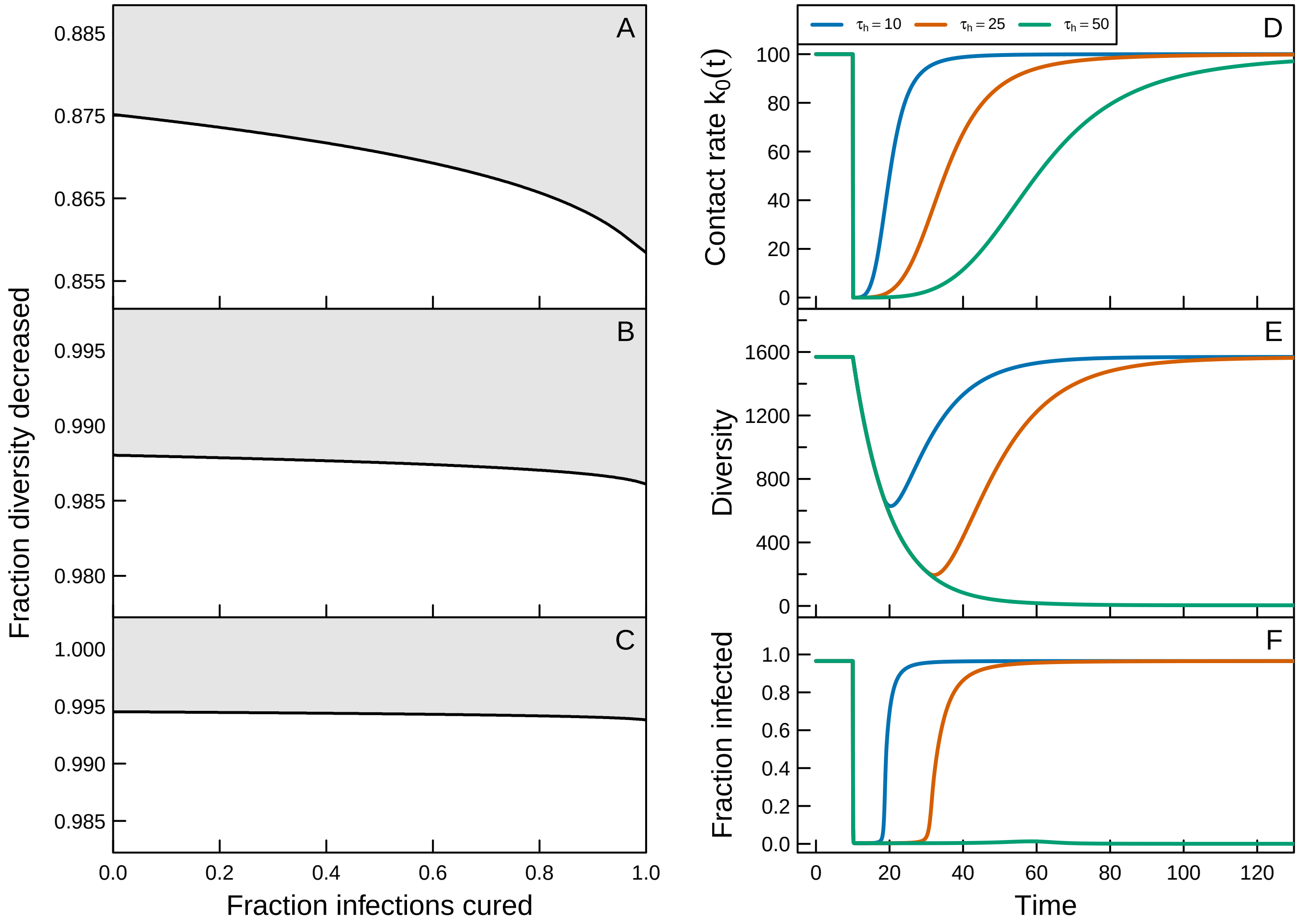

Fig. S4. Combinations of the fraction of infected individuals to be cured and the reduction in diversity that is required to change the population from the high-prevalence state to the low-prevalence state for transmission intensities $k_0 = 40, 70$ and 100 (*A-C*, respectively) in the simplified malaria model in terms of ordinary differential equations $\mathrm{Eq.}$ (S17) when the probability that an immigration event leads to the introduction of a new parasite gene, P_I, equals $2 \cdot 10^{-5}$ (see Figure S3, bottom-left). *Right*: Changes in the contact rate $k_0(t)$ (*D*), parasite gene pool diversity (*E*) and the fraction of the individuals that are infected (*F*) following a temporary and transient reduction in transmission potential, described by the time-dependent function $k_0(t) = 100\left((t-10)/\tau_h\right)^4/(1+((t-10)/\tau_h)^4)$.

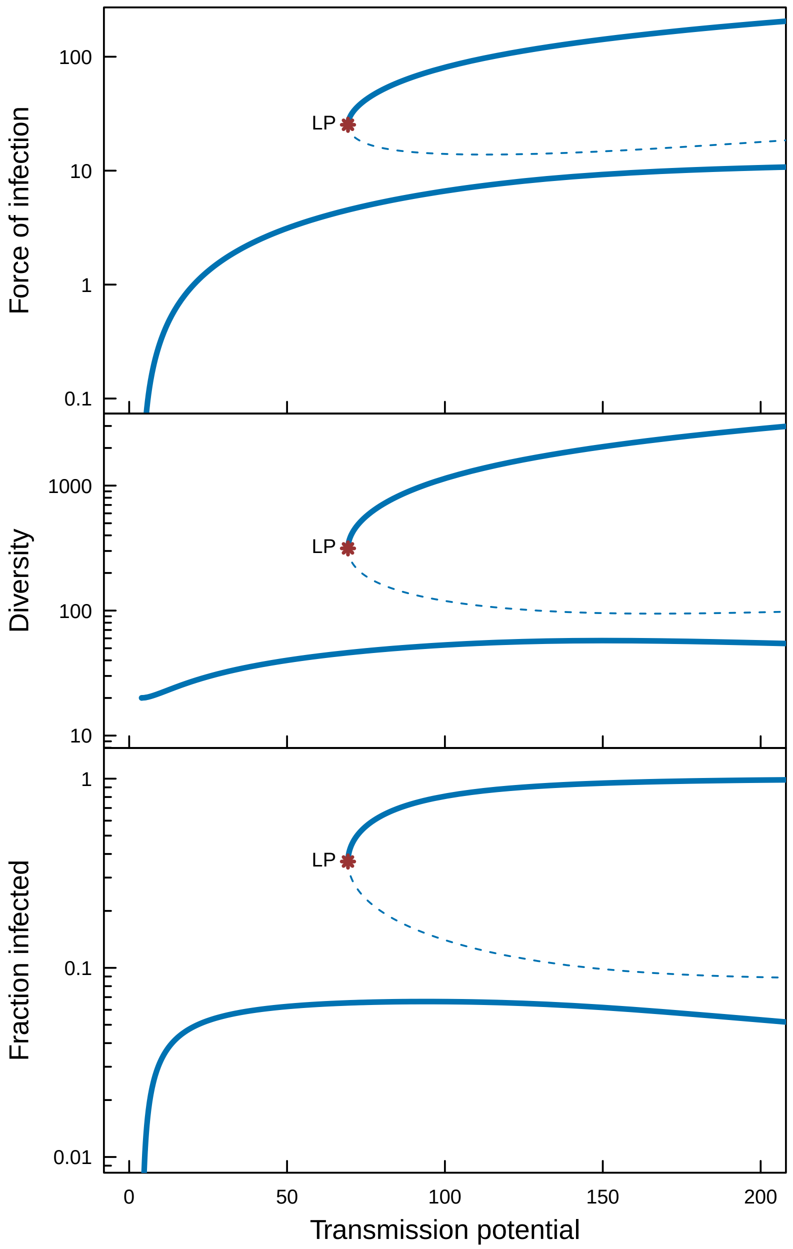

Fig. S5. Equilibrium states of the malaria model as a function of the transmission potential, the contact rate parameter k_0, for a closed population (no immigration: $\lambda_I = 0$, $P_I = 0$), in which the specific turn-over rate of pathogen genes is diversity-dependent and equal to $\delta \exp(1 - D/D_{min})$ with $D_{min} = 20$. Solid and dashed lines refer to stable and unstable equilibrium states, respectively. The location of the tipping point (saddle-node bifurcation point) is marked as LP. Transcritical bifurcation points satisfying the condition $R_0 = 1$ are invisible because of the logarithmic scale of the y-axes (see Fig. S6).

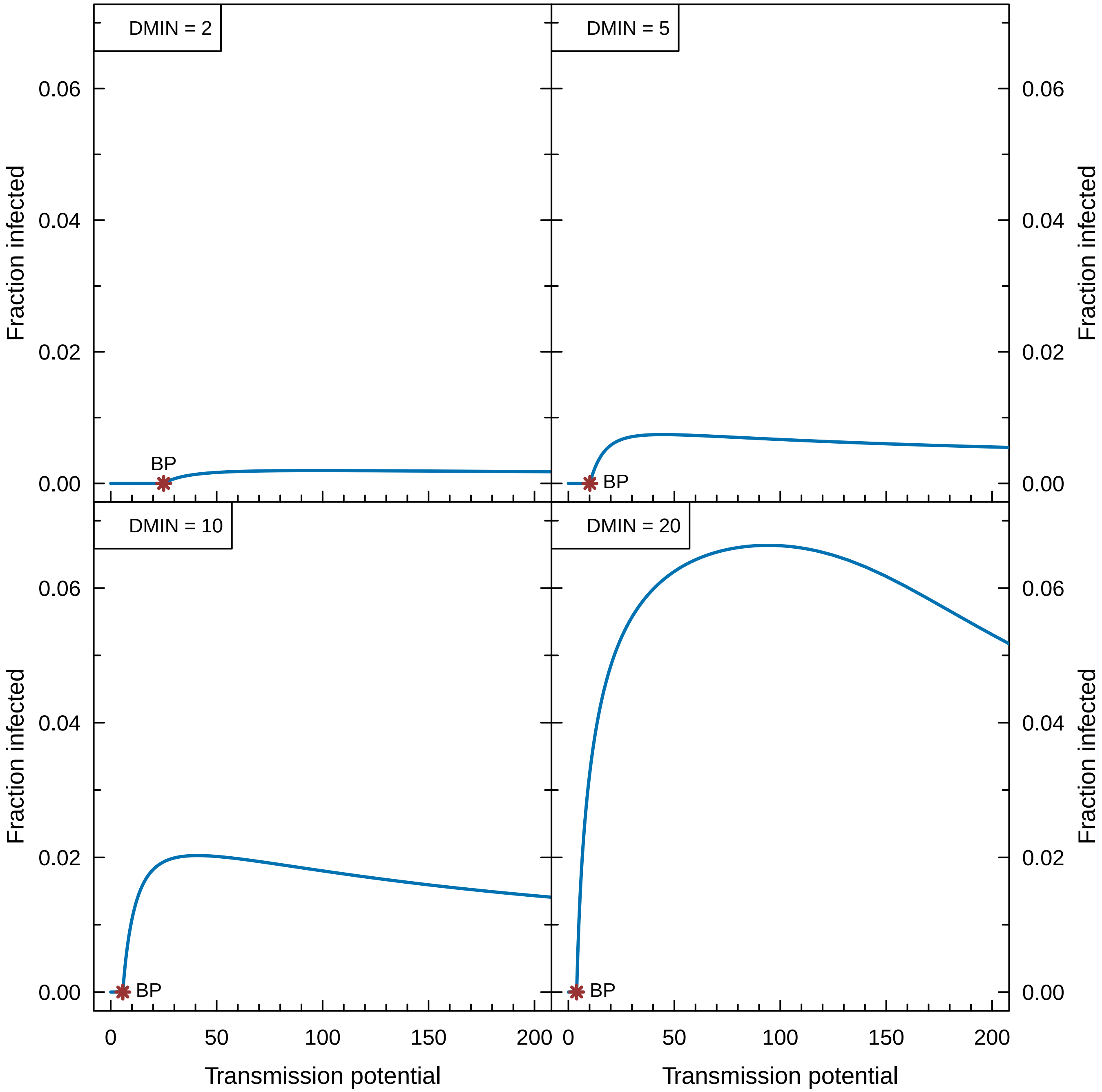

Fig. S6. Fraction of infected hosts in the equilibrium state of the malaria model as a function of the transmission potential, the contact rate parameter k_0, for a closed population (no immigration: $\lambda_I = 0$, $P_I = 0$), in which the specific turn-over rate of pathogen genes is diversity-dependent and equal to $\delta \exp(1 - D/D_{min})$ for different values of D_{min}. The locations of the transcritical bifurcation point satisfying the condition $R_0 = 1$ are marked as BP. Because of the scaling of the y-axes only the stable, low-diversity equilibrium is visible.

Table S1. Model parameters for the simplified ODE model.

Parameter	Value	Parameter	Value	Parameter	Value
N	10000	k_0	100	c_0	0.02
μ	0.02	L	20.0	p	0.9
δ	0.1	λ_I	1000	P_I	$5.0 \cdot 10^{-5}$
α	$6.8 \cdot 10^{-5}$				

References

1. R Águas, LJ White, RW Snow, MGM Gomes, Prospects for malaria eradication in Sub-Saharan Africa. *PLOS ONE* **3**, e1767 (2008).
2. D Alonso, A Dobson, M Pascual, Critical transitions in malaria transmission models are consistently generated by superinfection. *Philos. Transactions Royal Soc. B* **374**, 20180275 (2019).
3. K Dietz, L Molineaux, A Thomas, A malaria model tested in the African savannah. *Bull. World Heal. Organ.* **50**, 347–357 (1974).
4. AMd Roos, JAJ Metz, E Evers, A Leipoldt, A size dependent predator-prey interaction: Who pursues whom? *J. Math. Biol.* **28**, 609 – 643 (1990).
5. Q He, M Pascual, An antigenic diversification threshold for falciparum malaria transmission at high endemicity. *PLOS Comput. Biol.* **17**, e1008729 (2021).
6. MA Kirkilionis, et al., Numerical continuation of equilibria of physiologically structured population models. I. Theory. *Math. Model. & Methods In Appl. Sci.* **11**, 1101–1127 (2001).
7. AM de Roos, *deBif: Bifurcation Analysis of Ordinary Differential Equation Systems*, (2022) R package version 0.1.6.
8. A Claessens, et al., Generation of antigenic diversity in *Plasmodium falciparum* by structured rearrangement of *var* genes during mitosis. *PLOS Genet.* **10**, e1004812 (2014).
9. N Takahata, A simple genealogical structure of strongly balanced allelic lines and trans-species evolution of polymorphism. *Proc. Natl. Acad. Sci.* **87**, 2419–2423 (1990).
